# A critical evaluation of “leakage” at the cochlear blood-stria-barrier and its functional significance

**DOI:** 10.3389/fnmol.2024.1368058

**Published:** 2024-02-29

**Authors:** Kevin K. Ohlemiller, Noël Dwyer, Veronica Henson, Kaela Fasman, Keiko Hirose

**Affiliations:** ^1^Department of Otolaryngology, Washington University School of Medicine, St. Louis, MO, United States; ^2^Program in Communication Sciences and Audiology, Washington University School of Medicine, St. Louis, MO, United States

**Keywords:** stria vascularis, endocochlear potential, blood-labyrinth barrier, endothelial cell, pericyte, perivascular macrophage, basement membrane, cochlear lateral wall

## Abstract

The blood-labyrinth-barrier (BLB) is a semipermeable boundary between the vasculature and three separate fluid spaces of the inner ear, the perilymph, the endolymph and the intrastrial space. An important component of the BLB is the blood-stria-barrier, which shepherds the passage of ions and metabolites from strial capillaries into the intrastrial space. Some investigators have reported increased “leakage” from these capillaries following certain experimental interventions, or in the presence of inflammation or genetic variants. This leakage is generally thought to be harmful to cochlear function, principally by lowering the endocochlear potential (EP). Here, we examine evidence for this dogma. We find that strial capillaries are not exclusive, and that the asserted detrimental influence of strial capillary leakage is often confounded by hair cell damage or intrinsic dysfunction of the stria. The vast majority of previous reports speculate about the influence of strial vascular barrier function on the EP without directly measuring the EP. We argue that strial capillary leakage is common across conditions and species, and does not significantly impact the EP or hearing thresholds, either on evidentiary or theoretical grounds. Instead, strial capillary endothelial cells and pericytes are dynamic and allow permeability of varying degrees in response to specific conditions. We present observations from mice and demonstrate that the mechanisms of strial capillary transport are heterogeneous and inconsistent among inbred strains.

## Introduction

Cochlear stria vascularis establishes the composition of cochlear endolymph and generates the endocochlear potential (EP), which provides most of the electrochemical drive to hair cell transduction currents. The intrastrial micro-environment appears critical to these functions, and includes the cochlea's most dense capillary beds, which support the high level of strial metabolic activity. Problems that alter strial capillary function might therefore contribute to dysregulation of endolymph and potentially impair EP generation. Our focus here is strictly strial capillary barrier function and its relation to the EP and hearing. We are not concerned with conditions that overtly damage or constrict strial capillaries, injure critical strial pumps and channels, or violate strial boundaries, all of which will certainly impact hearing. A prevailing opinion is that strial capillary leakage can dissipate voltage or ionic gradients across capillary walls necessary to support the EP. An open-timeframe Google Scholar search using the terms “stria vascularis,” “capillary,” and “leak” or “leakage” revealed 1,630 papers (as of February 2024), while adding “endocochlear potential” or “endolymphatic potential” to the search terms reduced the sample to 518 papers. These ostensibly represent papers that relate vascular permeability to the EP. Based on all permutations of added key words “EP,” “endocochlear potential,” or endolymphatic potential” plus “methods” or “recording,” only 25 papers reported EP values in the context of capillary leakage. To us, that suggests that the great majority of papers have left key questions of causality untested, leading to speculation and theories that remain unproven. We present evidence that essential aspects of strial capillary transport—selectivity and transport mechanisms—vary across and within species. We suggest that strial capillary endothelial cells and pericytes function together to modulate traffic across the capillary endothelial barrier which is a dynamic and adaptive process by design.

## What cells and tissues do strial capillaries support?

The operation of the mammalian cochlea is energetically demanding, yet hair cells and cochlear neurons are located much further from their blood supply than are typical cortical neurons of the brain (10–20 μm vs. 50–100 μm for OHCs) (Schlageter et al., 1999). With regard to the organ of Corti, this arrangement is generally attributed to potential mechanical interference with hair cells by pulsating capillaries (Wangemann and Marcus, 2017). The nearest capillary to the organ, the vessel of the basilar membrane, is patchy or not patent in many species (Axelsson, 1988). Evolution has solved the blood supply problem by extensive use of anaerobic glycolysis by hair cells (Matschinsky and Thalmann, 1967), by potentially recruiting multiple capillary beds, and by relocating the most energetically intensive process to the stria vascularis. The stria creates most of the electrochemical force for transduction using a series of pumps and channels that move K+ up its voltage and concentration gradients, simultaneously creating high K+ endolymph that is also highly positively charged (Wangemann and Marcus, 2017). As a result, hair cells need only passively gate the flow of K+ through their soma and into the surrounding perilymphatic spaces. A further evolutionary innovation is that K+ is then “recycled” through the lateral organ of Corti and ultimately back to the stria via the spiral ligament (Wangemann and Marcus, 2017). In the ligament, K+ passes through outer sulcus cells, is actively taken up by Type II fibrocytes, then passed to Type I fibrocytes behind the stria through a Connexin 26 and 30 gap junctional network. The Type I fibrocytes are also directly coupled to strial basal cells, which in turn are coupled to strial intermediate cells through gap junctions. In gerbils the syncytium potentially extends to the endothelial cells surrounding the strial capillaries (Takeuchi and Ando, 1998), although this may not be the case for all mammals (Cohen-Salmon et al., 2007). That such a striking organizational principle of the stria may vary across species presents a cautionary note to generalizations about strial capillaries—a major theme of this review. If strial intermediate cells and endothelial cells are part of the same syncytium, then all cellular elements of the stria except for marginal cells appear best suited for two-way trafficking with the spiral ligament, leaving the marginal cells the sole arbiter of whether molecules from the intrastrial fluid reach the endolymph.

While the term “blood-labyrinth barrier” (BLB) dominates the literature, it does not specify any particular location or barrier (Sun and Wang, 2015; Salt and Hirose, 2018). It furthermore conflates divergent properties among capillary beds, their recipient tissues (stria, neurons, organ of Corti), and their surrounding fluids (endolymph, perilymph, intrastrial fluid). Gross scanning methods such as MRI (e.g., Floc'h et al., 2014; Veiga et al., 2021; Zhang et al., 2023) have been applied to diagnose “BLB leakage”, but do not indicate capillary sources. Blood-born metabolites could reach the organ of Corti via capillaries in the spiral ligament, indirectly via the stria, spiral limbus (Firbas et al., 1981), or the osseous spiral lamina. An early paper by Lawrence (1974) argued that neither strial capillaries nor spiral ligament capillaries play a part in maintaining the organ of Corti. A more recent study, however, identified a pathway for glucose that may begin in strial capillaries and extend to the lateral organ of Corti via the spiral ligament (Chang et al., 2008). In any case, the metabolites are delivered to the perilymph (Okumura, 1970; Ferrary et al., 1987; Ando et al., 2008), so that it is the aggregate permeability of all these that is determined by assaying the perilymph itself (Hirose et al., 2014a). The EP can be maintained for a period of time when the vasculature is perfused with K+-free and glucose-free media (Wangemann and Marcus, 2017). This indicates that strial K+, glucose, and presumably other metabolites are derived from the perilymph that bathes the spiral ligament. Ironically then, strial capillaries may not even deliver much of the glucose required by the stria (Ando et al., 2008; Hishikawa et al., 2015). The question then becomes “What cells, other than the stria itself, do strial capillaries supply?” The ionic composition of endolymph is determined by ion pumps and exchangers of the stria vascularis with contributions from Reissner's membrane and the inferior ligament (Muñoz et al., 2001). However, the literature provides no clear evidence that strial vessels supply oxygen or other nutrients to the organ of Corti via the endolymph. If strial capillaries are highly permeable, as we demonstrate in later figures, then the real limiting barrier for transfer of ions and metabolites to the endolymph is the transport selectivity of marginal cells. Vital though this function must be, relatively little is really known about marginal cell transporters. As prominent exceptions, megalin (LRP2) and cubilin, transporters located on the luminal side of marginal cells, appear involved in aminoglycoside trafficking in hair cells (Hosokawa et al., 2018; Kim and Ricci, 2022).

A long-standing belief regarding the microvasculature of the brain, cochlea, and retina was that these specialized vascular networks evolved to prevent the entry of pathogens and inflammatory cells into these “privileged” spaces due to the risk of bystander injury. The phrase “immune privilege” was commonly used to characterize the special environment created by the blood-brain barrier, the blood-labyrinth barrier, or blood-retinal barrier. This view was challenged, however, by the discovery of robust monocyte and macrophage infiltration following stressors such as noise exposure and aminoglycosides, both of which are non-infectious stimuli (Hirose et al., 2005, 2014b; Tornabene et al., 2006; Sato et al., 2010; Bae et al., 2021). Cochlear macrophages accumulate in the inferior spiral ligament, then migrate to other positions in the ligament, as well as surfaces lining cochlear scala tympani. Surprisingly, this infiltration largely spares the stria, which flies in the face of the general perception that spiral ligament capillaries appear “tighter” than strial capillaries. If there were no immune-privilege, the marginal cells would still pose a significant barrier to either macrophages or their secretions entering the endolymph. Strial capillary architecture may have been driven primarily by a requirement to protect and support strial constituent cells.

If circulating mononuclear phagocytes cannot gain entry into the intrastrial space, then waste and cell debris must be managed by resident cells of the stria. In the central nervous system, hydrostatic and oncotic pressure favor net flow of water from capillaries into interstitial fluid (Groothuis et al., 2007). This “bulk flow” carries a host of small molecules. Cellular waste, proteins, and excess water are typically removed where capillaries and post-capillary venules meet. From this, there can be extensive water flow radiating outward and along the capillary path. What is not absorbed by the venous system may be resorbed by lymphatic vessels that have been reported in the spiral ligament (Keithley, 2022), although the evidence for lymphatic vessels in the spiral ligament is limited. The interior of the tightly sealed strial space (see below) may be limited by this standard arrangement, in that (1) longitudinal water flow within the stria may not be permissible, (2) the post-capillary venules of the inferior ligament may be too far away, (3) the largely uncharacterized lymphatics of the spiral ligament may be too remote and may have poor access to intrastrial fluid, and (4) the post-capillary venules in the ligament have been found to have an elevated hydrostatic pressure, which could discourage re-entry of water and waste products (Shaddock et al., 1985). In addition to myriad waste products, debris from dying cells must be removed. The stria vascularis is populated by resident macrophages that are present from early in development and are typically located in the perivascular space (Shi, 2010). These resident macrophages are a non-exchanging population and undergo marked transformation in morphology over time. They appear to remain in the intrastrial space over the lifetime of the animal (Noble et al., 2021).

## Normal strial operation

The stria forms a “sandwich” structure, bounded laterally by basal cells and medially by marginal cells. Adjacent cells in these layers are bound to each other by tight junctions, such that the intrastrial space is isolated from the adjacent endolymph and perilymph without free flow of water or ions. The proximate event to EP generation is taken to be a large K+ efflux current through Kir4.1 channels in the strial intermediate cell membrane (Takeuchi et al., 2001; Wangemann and Marcus, 2017). The K+ is then removed from the interstitial (intrastrial) space by Na+/K+ ATPase and Na+/K+/Cl- co-transporters into strial marginal cells, and ultimately released in a highly regulated manner back into scala media by KCNQ1/KCNE1 channel assemblages. Because marginal cells are not electrically connected to other strial cell types, they must actively or passively transport K+ and other key components of the endolymph.

**Figure 1:**
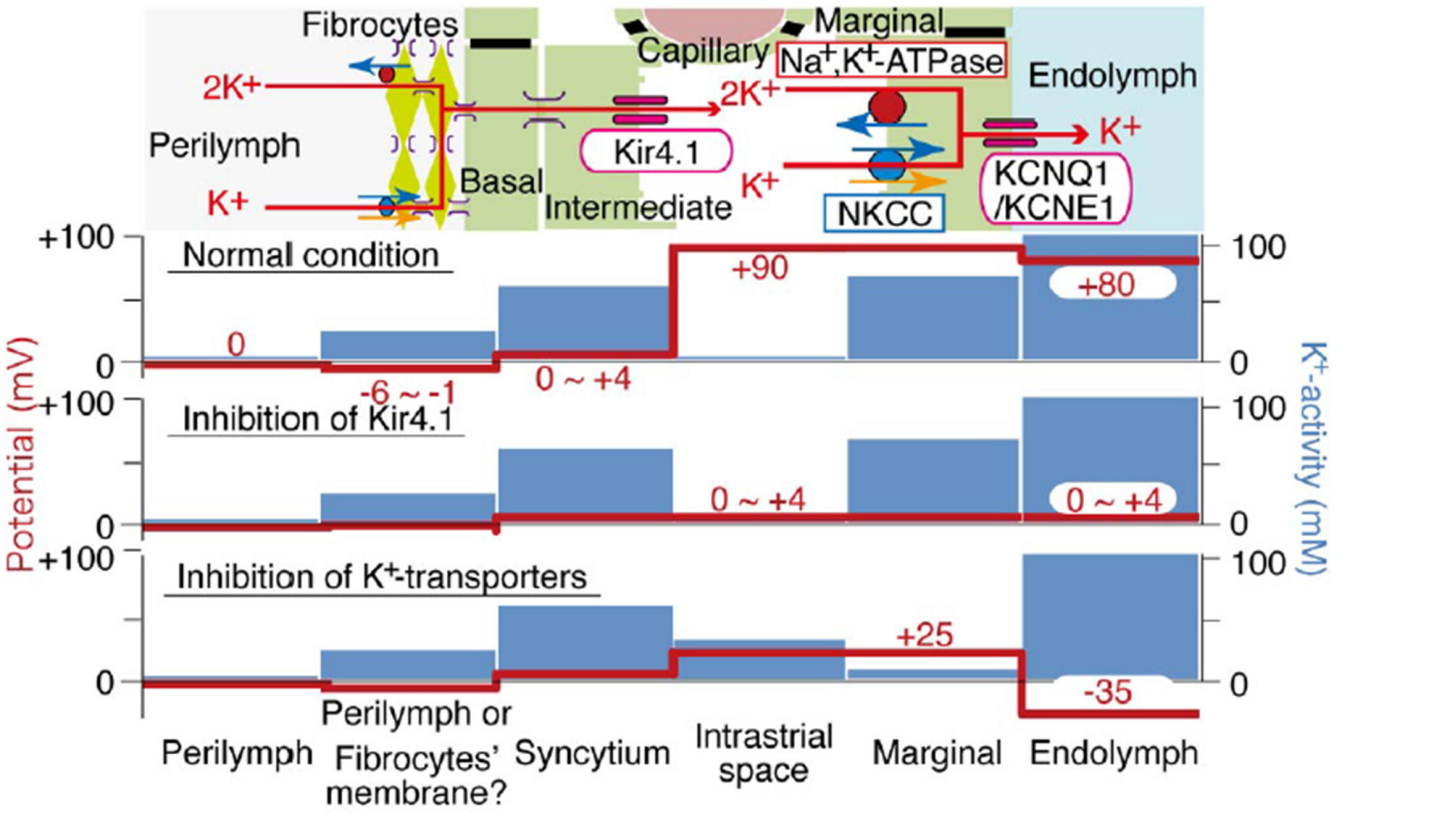
Schematic of electrochemical properties of the lateral cochlear wall. Upper panel shows the structure of the lateral wall and the K+ transport apparatus involved in the generation of the EP. The predicted voltage and K+ concentration in each compartment under normal conditions vs. inhibition of Kir4.1 and strial K+ transporters are respectively shown in other images (from Nin et al., 2008, with permission).

The movement of K+ down its concentration gradient from strial intermediate cells into the intrastrial fluid also generates a high positive voltage within the stria. Further, because of the large net positive current flowing into marginal cells, these cells are likewise positively charged. Thus, EP and endolymph generation are both inextricably linked to large positive voltages in the intrastrial space and within marginal cells. These typically exceed the EP itself. Marginal cells must take up K+ directly from the intrastrial space against a steep concentration gradient, and failure to keep intrastrial K+ at a very low level interferes with both endolymph production and EP generation. Notably, however, the dependence of the EP on endolymphatic K+ appears not as steep as does the dependence on intrastrial K+, so that a reduced EP may be observed even with normal endolymph K+ concentration (Schmiedt, 1996). Figure 1 shows the K+ concentration and electrical potential of cells and compartments of the stria under normal and pathologic conditions. Note that the most significant differences between the “normal” and “abnormal” cases correspond to failure to deliver K+ to the intrastrial space (Kir4.1 inhibition) or failure to remove it (K+ transporter inhibition).

The use of K+ as a primary excitatory current carrier in mechanotransduction is highly unusual in all of neurophysiology. In theory, hair cell depolarization could be carried by the Na+ current, as in neurons. The use of Na+ would likely have required that hair cells express the Na+/K+ ATPase, which would expose them to greater metabolic stress. Another benefit of utilizing K+ instead of Na+ may be the stoichiometry of the Na+/K+ ATPase, which moves three K+ ions into strial marginal cells for every two Na+ ions out. The standing currents in scala media are quite large (188–376 pA per marginal cell) (Zidanic and Brownell, 1990) relative to, say retina (70 pA per retinal rod), and 50% more K+ can be mobilized—and removed—relative to Na+, for the same energy expenditure.

## Transport modes of strial capillaries

**Figure 2:**
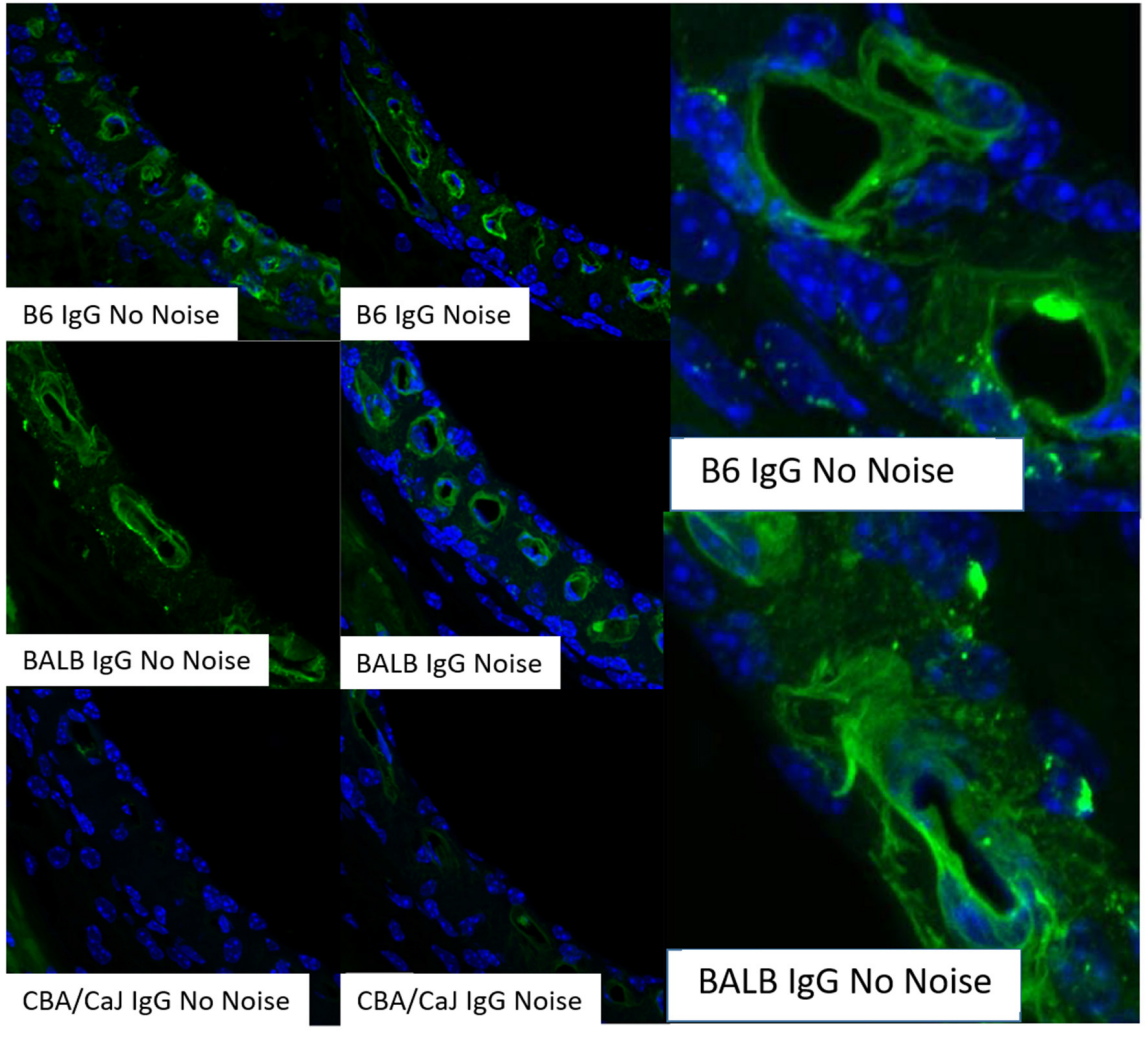
Strial perivascular appearance of innate IgG depends on inbred strain. Radial confocal views of innate IgG antibody staining in lower basal stria vascularis in C57BL/6J (B6), BALB/cJ (BALB), and CBA/CaJ inbred mice with, and without, noise exposure (4.0–45.0 kHz, 110 dB SPL, 2 h). Exposed animals were examined immediately after exposure. Results shown are typical of five replicates per strain and condition. Panels at right show enlarged view of different BALB and B6 no-noise replicates. DAPI (blue) not shown in all cases (adapted from Dwyer, 2010; see same for Methods section).

Most capillaries in the body share a basic structure consisting of a single sheet of endothelial cells rolled into a cylindrical tube, surrounded by basement membrane with pericytes and perivascular macrophages within close proximity (Groothuis et al., 2007). These cells jointly govern cellular and molecular traffic in and out of capillaries. Capillary endothelial cells are joined by tight and adherens junctions, whose specific molecular makeup can vary by tissue. Accordingly, the “tightness” of inter-endothelial junctions can vary also. Ions, proteins, and metabolites exit capillaries by passing either through (transcellular transport) or around (paracellular transport) the capillary endothelial cells. Both of these represent normal modes of operation, and neither can be treated as inherently more pathological. Moreover, there is evidence that endothelial cells can switch pathways if one is inhibited (Armstrong et al., 2012; Muradashvili et al., 2012). Transcellular transport encompasses several processes, including carrier-mediated transport, receptor-mediated transport, and adsorption-mediated transport. Some of these operate bi-directionally. Paracellular pathways in peripheral capillaries typically favor low molecular weights and particle diameters < 5 nm (Rabanel et al., 2012). This includes small ions, water, and a host of small molecules that include popular tracers such as fluorescein and small fluorescein-conjugated dextrans (Saunders et al., 2015) (Table 1). Small proteins such as albumin, horseradish peroxidase, and myeloperoxidase span the size limit between paracellular and transcellular transport, but tend toward transcellular transport in strial capillaries (see below). The conditions under which large proteins such as IgG are transported are not clear, but this protein appears to have its own receptors for transcellular transport (Zlokovic et al., 1990). The presence of IgG in the interstitium has generally been interpreted to indicate pathology (see below). However, we observe it under normal conditions in mice in a manner that appears strain-dependent and non-pathological. Figure 2 shows IgG staining in the stria of C57BL/6J (B6) and BALB/cJ (BALB), but not CBA/CaJ mice. The extent and pattern of staining are unchanged by noise exposure. In B6 and BALB mice, most of the IgG appears trapped in the capillary basement membrane, although there are also indications within the intrastrial space (Figure 2, right panels). The transport of IgG may be adaptive, or may be incidental to some other adaptive process. Such a striking strain difference in what might be expected to be a conserved and fundamental process highlights the value of inbred mouse models, and suggests that we do not yet know the factors shaping strial capillary transport. Albumin, the most abundant plasma protein, constantly crosses the endothelial barrier in both directions, acting as a chaperone for a number of hydrophobic molecules. From this, one would expect to find albumin in the interstitium under normal circumstances. Evans Blue (EB) is a small molecule often used as a vascular tracer. Based on its size, it would be anticipated to be transported paracellularly. However, EB in capillaries is bound by albumin, so that its movement is typically transcellular (Patterson et al., 1992).

**Table 1:** Sizes of commonly-used molecular tracers.

	**Molecular weight (kDa)**	**Radius (nm)**	**Probable route of transport**
Na fluorescein	0.376	NR	Paracellular
Evans blue^*^	0.96	NR	Transcellular
Horseradish peroxidase	≈44	3	Transcellular
Albumin (unlabeled)	69	3.5	Transcellular
IgG	≈155	5.3	Transcellular
Dextrans	1.5–2,000	0.8–38.2	Depends

Modified from Saunders et al. (2015).

* Typically bound to albumin.

Pericytes appear primarily responsible for directing endothelial cell traffic (Armulik et al., 2005; Shi et al., 2008; Geevarghese and Herman, 2014; Attwell et al., 2016; Shi, 2023), and it is likely they determine what transport modes and what types of bi-directional traffic are active at any time. Pericytes are important for the development and survival of capillaries, as well as capillary branch formation, and loss of pericytes can lead to capillary closure and loss. Genetic ablation of strial pericytes in mice (Zhang et al., 2021; Shi, 2023) leads to strial capillary constriction and loss, EP reduction, hair cell loss and hearing loss. However, such ablations have been universal, so that deficiencies in multiple capillary beds may have driven these results. The ratio of pericytes to endothelial cells is also a significant variable, such that a higher ratio corresponds to less transport (Daneman et al., 2010). The roughly 1:1 ratio in strial capillaries is similar to brain and retina (Shi et al., 2008). Other players not yet considered include the capillary basement membrane and perivascular macrophages. Basement membranes are sugar and collagen extracellular matrix structures that surround most capillaries in the body (Kottke and Walters, 2016). They line the abluminal side of endothelial cells, but surround the pericytes, and are maintained by both cell types. In addition to demarcating developing tissue boundaries and promoting development-related cell movement, they act as filters to slow movement of some molecules, and discourage the entry of inflammatory cells. In strial capillaries, the basement membrane can become thickened during aging (Thomopoulos et al., 1997; Suzuki et al., 2016), Type 2 diabetes (Williamson et al., 1973), and Alport syndrome (Gratton et al., 2005). Perhaps somewhat non-intuitively, thickened basement membranes are associated with increases in vascular permeability (Kottke and Walters, 2016). While such changes in strial capillaries are likely to represent pathology, specific evidence of their role in hearing and deafness is lacking.

Perivascular macrophages (PVMs) are reported in brain, and may have a parallel in the perivascular macrophage/melanocytes (PVM/Ms) of the stria suggested by Shi et al. (Zhang et al., 2012, 2013). However, the melanocyte function of PVM/Ms has been disputed (Hirose and Li, 2019; Ito et al., 2022), and these cells more likely correspond to the resident PVMs in other tissues. Strial melanin is thought to originate from the influx during development of melanocytes that give rise to intermediate cells (Steel and Barkway, 1989). Over time, all three major cell types of the stria (marginal, basal, intermediate) can take up melanin granules (Wright and Lee, 1989; Hayashi et al., 2007; Ohlemiller et al., 2009), potentially muddying the identity and purpose of cells containing melanin. Earlier studies identified apparently pigmented cells bordering capillaries that were distinct from intermediate cells (Conlee et al., 1989), and multiple authors have suggested the existence of two or more types of intermediate cells (Cable and Steel, 1991; Conlee et al., 1994; Spicer and Schulte, 2005a). Single-cell transcriptomics, which might help establish the number of sub-types of strial melanocytes have yet to identify distinct populations (Taukulis et al., 2021; Boussaty et al., 2023). As we pointed out, waste clearance in the stria may be particularly challenging and place a premium on local macrophage function. We therefore favor the interpretation that PVM/Ms are simply resident strial macrophages whose job is to transfer waste back to strial capillaries. That said, it is not clear from published ultrastructure that strial PVMs form the necessary junctions with capillaries to support this function. Although there are claims that genetic ablation of PVMs promotes strial capillary leakage, hearing loss and EP reduction (Zhang et al., 2012), the universal elimination of PVMs in these experiments could have exerted additional effects beyond the stria. Moreover, other mouse KO models for cochlear macrophages that would have eliminated the same cells (Hirose and Li, 2019) showed no hearing loss or EP reduction. From this evidence it cannot be concluded that strial PVM ablation and PVM-related capillary leakage cause hearing loss and EP reduction. It would not be surprising, moreover, if impairment of strial waste collection should be problematic for strial function overall.

**Figure 3:**
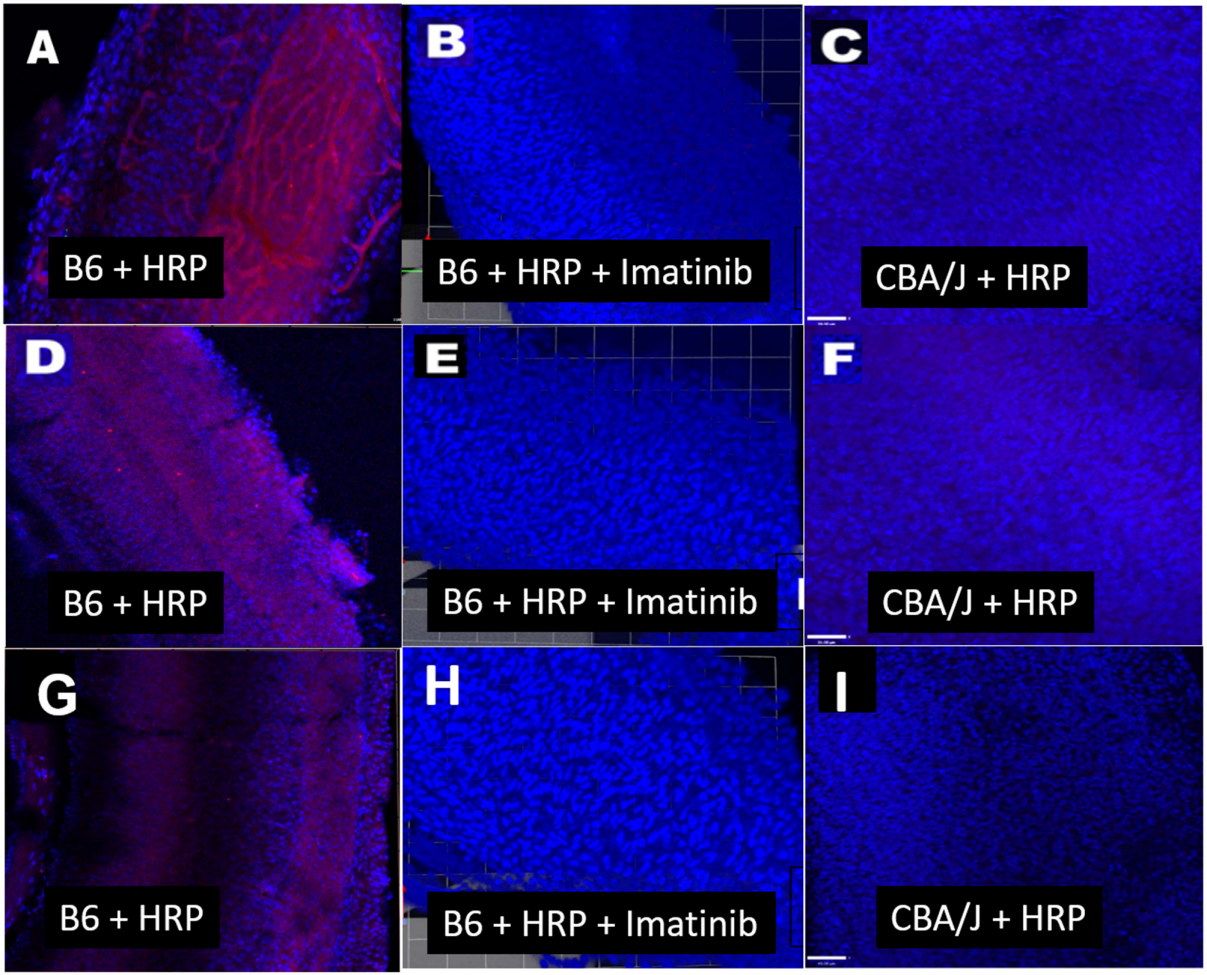
Transcellular strial capillary transport of HRP depends on inbred strain. Confocal views of three replicate surface preps of stria vascularis showing application of fluorescent HRP in C57BL/6J (B6) mice **(A, D, G)**, co-application of HRP and Imatinib in B6 mice **(B, E, H)**, and application of HRP in CBA/J mice **(C, F, I)**. Compounds were applied trans-cardially (adapted from Henson, 2013; see same for Methods section).

**Figure 4:**
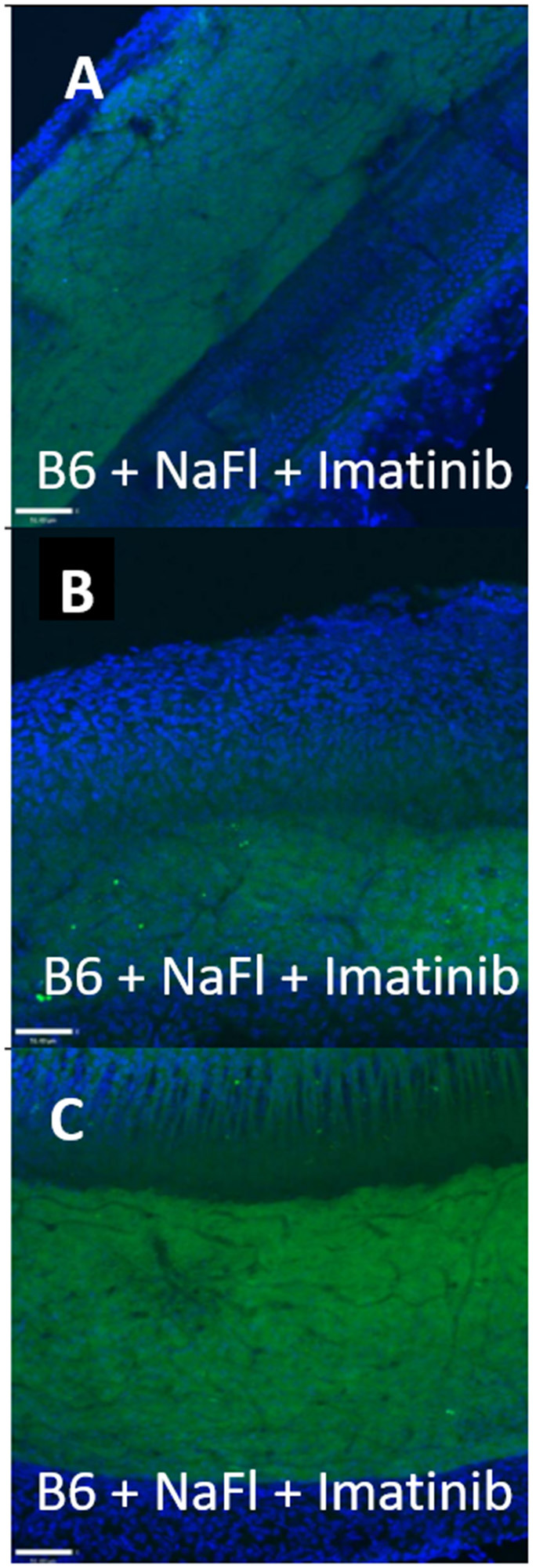
Strial capillary transport of NaFl in B6 is paracellular. **(A–C)** Confocal views of three replicate surface preps of stria vascularis showing trans-cardial co-application of NaFl and Imatinib in C57BL/6J (B6) mice (adapted from Henson, 2013; see same for Methods section).

The mode of capillary transport in a given system can be probed using several approaches. Transmission electron microscopy can show the lack of tight junctions between endothelial cells, but most such evidence is anecdotal and not quantitative. Tyrosine kinase blockers of trans-endothelial transport, such as Imatinib, can be applied to test for transport processes (Coffin et al., 2021), as we also show here (Figures 3, 4). *In vivo* and *in vitro* studies fluorescent tracer studies can establish whether capillary leakage is occurring and suggest the mode, based on size. *In vitro* tests involve establishing a confluent sheet of endothelial cells on a porous membrane and assaying either the transfer of fluorescent signal across the sheet or the trans-endothelial electrical resistance (TEER) across it. The endothelial sheets can be chemically manipulated or supplemented with other cells (e.g., pericytes, PVMs) to test their effect on permeability (Neng et al., 2013). Reported maximum TEER estimates for strial capillary endothelial monolayers have varied widely, ranging from ~40 to 200 Ohms/cm^2^ (Sekulic-Jablanovic et al., 2022; Sekulic et al., 2023), so that it appears not yet possible to compare strial and brain capillaries using this method. Tests for fluorescent tracer movement likely assess both trans-cellular and paracellular leakage, while direct electrical measurements may be specific for paracellular leakage (Table 1). Neither permit the tracking of molecules *into* strial capillaries, so that half of a probable two-way conversation remains uncharacterized. Notably, *in vitr*o barrier diffusion experiments in endothelial cell sheets (Neng et al., 2013, Figure 5B; but see Zhang et al., 2012, Figure 2F for contradictory data) indicate that the addition of pericytes and PVMs add only marginally to the tightness of strial capillary transcellular and paracellular barrier function, raising questions about the true impact of these cells. We really know relatively little about the specific transport mechanisms of strial capillaries. There are no comparison RNAseq data for strial vs. retina or brain endothelial cells and pericytes to suggest whether these cells operate uniquely in the stria. Clearly, capillaries are in the transport business, as how else can they supply surrounding tissues? But when and where does it become pathological, and is it more likely to apply to trans-cellular or paracellular processes?

## Normal strial transport: previous observations

**Figure 5:**
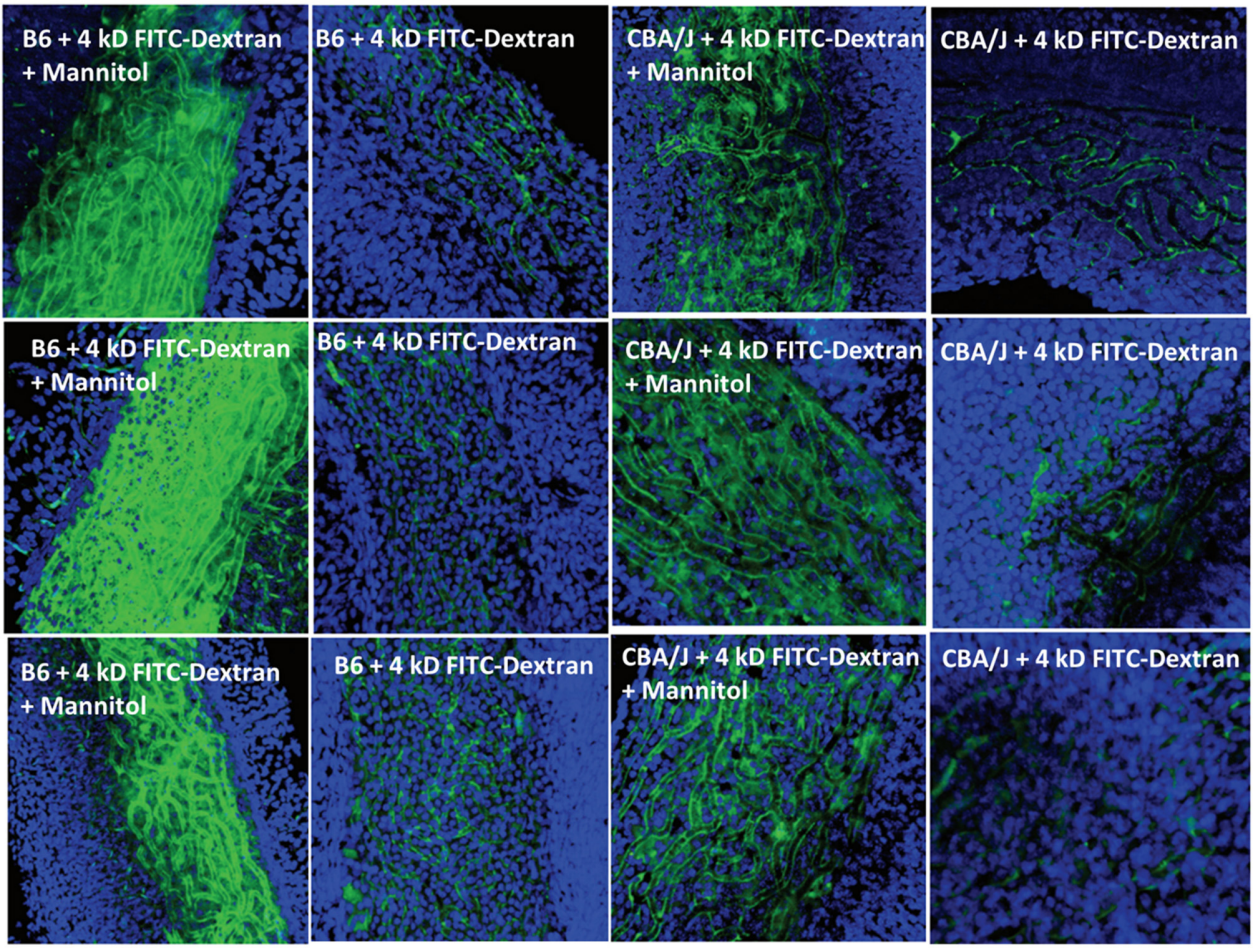
Effects of mannitol in promoting paracellular transport of 4 kD FITC-dextran depend on inbred strain. Confocal views of surface preps of stria vascularis showing three replicates of trans-cardial 4 kDa FITC-dextran without co-administration of mannitol, or 1 h after IP mannitol, in C57BL/6J (B6) and CBA/J mice (adapted from Fahrenthold, 2015; see same for Methods section).

**Figure 6:**
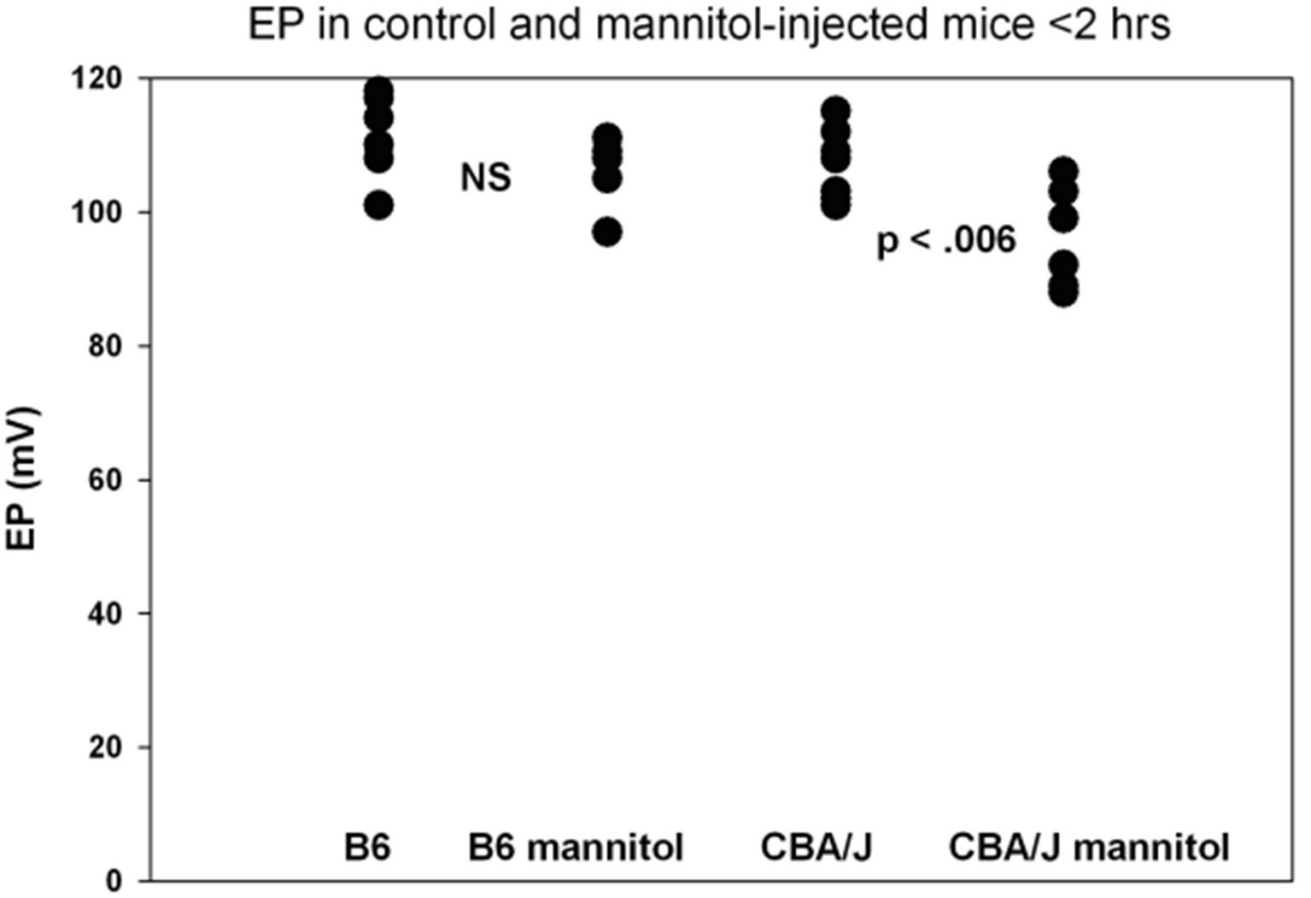
Functional effects of mannitol are mild and depend on inbred strain. Basal turn EP measures for mice treated as in Figure 5 within 2 h of IP mannitol administration (*n* = 5–8 per strain and condition). Means for each group were 111.3, 106.0, 106.4, and 96.2 mV, respectively. The range for mannitol-treated CBA/Js was 88–106 mV (adapted from Fahrenthold, 2015; see same for Methods section).

A host of studies going back 50 years have addressed the transport properties of strial capillaries in chinchillas, guinea pigs, and mice under standard conditions (e.g., Duvall et al., 1971, 1979; Osako and Hilding, 1971; Gorgas and Jahnke, 1974; Sakagami et al., 1982a,b, 1987; Duvall and Klinkner, 1983; Xu et al., 1994). These have tracked exogenous horseradish peroxidase (HRP), myeloperoxidase (MRP) and other tracers at the light or electron microscope level. Most have been somewhat qualitative. Such studies have consistently painted a picture of fairly unrestricted passage of tracers into the intrastrial space via trans-endothelial transport. The amount of transport may be increased by noise exposure and other manipulations (see below). Our own experiments in inbred mice (Figures 2–5) paint a somewhat nuanced picture of capillary transport that varies by strain. Figure 3 suggests strain differences with regard to the transport of HRP, whereby B6 mouse stria readily allows passage of this foreign protein, while CBA/J mice do not. Moderate-sized proteins would be expected to pass from capillaries via a transcellular mode (Table 1), and accordingly, transport of HRP in B6 mice can be inhibited by Imatinib. By contrast, CBA/J mice do not show transport of HRP, even without Imatinib. This indicates that these two mouse strains possess very different selectivities for trans-endothelial traffic, and one would obtain very different impressions by just studying one of these strains. Figure 4 confirms that B6 strial capillary transport of sodium fluorescein (NaFl) is not affected by Imatinib, as expected for this small molecule tracer (Table 1). Figure 5 again indicates more restrictive transport in CBA/J mice. In this case, treatment with IP mannitol 1 h before sacrifice led to the dispersion of 4 kDa FITC-dextran in both B6 and CBA/J mice, but to very different extents. Without mannitol, neither strain showed passage of this tracer into the stria. Mannitol and glycerol are osmotic disruptors applied clinically to diagnose and treat Meniere's disease and sudden-onset hearing loss (Stahle, 1984; Filipo et al., 1997). They are thought to promote paracellular transport by distorting capillary endothelial cells (Le and Blakley, 2017). Therefore, these strain differences in Figure 5 appear in the context of paracellular transport. Overall, these results indicate that both transcellular and paracellular strial capillary transport mechanisms differ by genetic background. Transport selectivities are not invariant, even within species, and there is no single “normal” phenotype. Figure 6 further shows that mannitol did subtly lower the EP in both B6 and CBA/J, statistically so in the latter, yet probably not enough to elevate thresholds. We know from other studies (Santi et al., 1985) that mannitol causes transient edema in the stria, likely by building up in the intrastrial space and drawing water along with it. It seems strange that such a dramatic disruption may have little or no deleterious effects on hearing, yet this contention is also supported by the lack of hearing loss among subjects treated with mannitol or glycerol (e.g., Yoshida and Uemura, 1991; Wood et al., 2014), and should not be surprising. In summary, the evidence supports only somewhat restricted passage of metabolites and proteins from strial capillaries into the intrastrial space, irrespective of transport mechanism. Depending on genetic background, both innate (IgG, albumin) and foreign proteins (HRP, MRP) may be transported under normal conditions, possibly in both directions. It remains a question why there may occur such free passage of proteins that do not clearly serve an adaptive purpose. More broadly, it is difficult to discern why such an elaborate machinery surrounding strial capillary transport (endothelial cells, pericytes, basement membranes, perivascular macrophages) should exist, yet not promote strict transport into a closed space with highly demanding requirements.

## Normal strial capillary transport: theory

Existing models for the origin of the EP share one important feature: Beyond supplying metabolites to the stria itself, strial capillaries do not contribute directly to the generation of the EP (Nin et al., 2008; Wangemann and Marcus, 2017). However, dysregulation of the vascular barrier between capillaries and the intrastrial space could potentially perturb the EP. We have emphasized that K+ levels must remain low in the intrastrial space to maintain a normal EP. We are therefore most concerned with abnormal K+ flux *into* the intrastrial space, while the reverse flow (dissipation of K+ into the capillary lumen from the interstitial fluid) is not favored. The electrical charge associated with strial capillaries is an important factor here. Strial basal and intermediate cells possess a low membrane voltage (0–4 mV), but a high K+ concentration (Figure 1). If these cells are electrically connected to the endothelial cells, then the latter would also have high K+ levels and a low membrane potential. In either case, the dominant ion in their cytoplasm will be K+. Any K+ transport out of strial capillary endothelial cells into the intrastrial space will be favored by the concentration gradient for K+, but opposed by the steep voltage gradient, so that overloading of the intrastrial fluid by K+ from endothelial cells seems unlikely. As for the capillary lumen itself, most blood proteins carry a negative charge, as do the red blood cells. This preponderance of negative charge will draw balancing positive charges in a probable free flux of water and small ions. Thus, we predict that the capillary lumen will have no net charge, or 0 mV. If strial capillary endothelial cells are *not* connected directly to intermediate cells, they are still predicted to have high K+ levels and a low membrane potential. Published values for endothelial cell membrane potential indicate a moderately negative charge of ~-50 mV (He and Curry, 1995), so that these cells would resemble most cells whose membrane voltage is dominated by the equilibrium potential for K+. The endothelial cells would thus be perched between the low-K+, high-voltage intrastrial fluid and the low-K+, low-voltage capillary lumen. In this scenario also, opposing gradients would discourage significant K+ net movement into intrastrial fluids. In sum, the concentration and voltage gradients across strial capillary walls do not favor significant net K+ movement into or out of strial capillaries. If there are other significant metabolites or mediators that are transported into the intrastrial space, and in so doing impair strial function, it is not clear what these are.

Many of the claims regarding the effects of abnormal strial capillary properties are based on pathological conditions such as aging, noise exposure, and inflammation. We examine those conditions in the following paragraphs.

## Strial barrier dysfunction in presbycusis

Schuknecht identified cases of human presbycusis that seemed to reflect primary pathology of the stria (Schuknecht, 1964; Schuknecht et al., 1974). Animal studies have led to Mongolian gerbils (Schulte and Schmiedt, 1992; Spicer and Schulte, 2005b) and several inbred mouse strains (Ohlemiller, 2009) as modeling the condition in ways that recapitulate key observations in humans. Humans and animals share an etiology that focuses on strial marginal cell loss and dysfunction, perhaps reflecting the high metabolic wear-and-tear in these cells. In addition, the stria becomes thinner, which corresponds to a loss of processes by strial constituent cells. The mouse studies also indicate a genetic predisposition, since only some mouse strains tend toward age-related EP reduction. B6 mice present an interesting case because they show fairly rapid hair cell loss with age, but their EP remains normal to the end of the lifespan (Ohlemiller et al., 2006). They do show loss of marginal cells with age, but apparently below any threshold for EP changes. They also show a rough doubling in the thickness of strial capillary basement membranes, which is also one of the hallmarks of the aging gerbil cochlea (Thomopoulos et al., 1997). In gerbils, this has been interpreted as an indicator of hypoperfusion (Gratton et al., 1995, 1996), and a similar effect—perhaps below some threshold effect—cannot be ruled out in the mice. Mouse and gerbil studies suggest a pattern of fewer, larger capillaries in older animals with a low EP (Gratton et al., 1997; Ohlemiller et al., 2006, 2010). Such capillary changes are consistent with reports of pericyte pathology in older mice (Neng et al., 2015). While strial hypoperfusion certainly appears possible with age, abnormal capillary permeability is not a theme of the literature in strial or other forms of presbycusis.

## Strial barrier dysfunction following noise exposure

The acute effects of noise exposure on the stria have been characterized by a number of authors over decades. An acute increase in strial capillary permeability is supported by several studies (e.g., Duvall et al., 1974; Hukee and Duvall, 1985; Duvall and Robinson, 1987; Goldwyn and Quirk, 1997; Suzuki et al., 2002; Shi, 2009a,b; Hou et al., 2018; Jiang et al., 2023). Most of these reports are consistent with the idea that the increase in permeability was due to an increase in transcellular transport (but see Wu et al., 2014). While several studies indicate that noise results in strial thinning and reduced capillary density (e.g., Hirose and Liberman, 2003; Hou et al., 2020), such changes typically coincide with complete EP recovery. Pericyte pathology that could promote strial capillary thinning and loss has been reported following intense noise in B6 mice (Shi, 2009a,b; Hou et al., 2018; Jiang et al., 2023), although no direct link to the EP or hearing was shown. A similar study in B6 mice by the same group (Hou et al., 2020) applied neonatal pericytes to the perilymph and reported improvements in the EP and thresholds, but the injected cells were not tracked, and could have lodged near the organ of Corti and exerted trophic effects. Pericytes broadly express trophic factors that could also act on the damaged organ of Corti. Another study in B6 mice from the same group attributed noise-induced hearing loss and loss of EP to pathology of PVM/Ms and impaired secretion of pigment epithelium growth factor (PEGF/PEDF) by these cells (Zhang et al., 2013). The changes corresponded to a loss of strial capillary barrier function against FITC-albumin, although likely through trans-endothelial transport, as seen after noise in other models. Systemic PEDF was shown to improve the EP and hearing thresholds. But PEDF is also found in the organ of Corti (Gleich and Piña, 2008) where it may also exert trophic effects. Notably, the mouse strain, age, and type of exposure used in these studies have been shown to produce massive disruption of the organ of Corti (Ohlemiller et al., 2018), and strial changes were likely not the major drivers of hearing loss. Studies that apply intense noise to animals, then try to interpret the sequelae solely in terms of strial barrier function risk overlooking organ of Corti, and even strial histopathology, as guides. It is further confusing when intrastrial leakage of tracers such as Evans blue, albumin, or IgG are inexplicably assumed to be paracellular in the study design (e.g., Zhang et al., 2012, 2013; Jiang et al., 2023). Such a focus should at least be explained, and seems to reflect an expectation that paracellular leakage is more likely after traumatic injury, when there really is no evidence for this.

Inbred mouse strains usefully differ with regard to both acute and permanent strial injury by noise. B6, BALB, and CBA/J mice show very different acute EP profiles vs. age, noise intensity, and duration (Ohlemiller et al., 2018). In young mice (6–7 weeks) these differences are isomorphic with a greater likelihood that the reticular lamina will be opened in BALB and CBA/J mice. In such cases, the open reticular lamina would dominate any primary effect of the noise on the stria. Nevertheless, it is young B6 mice that show permanent collapse of the EP (<10 mV) after exposure to intense noise, which collapse accords with a prominent disruption of ZO-1 in the reticular lamina (Ohlemiller et al., 2018). In addition, a survey of recombinant inbred strains formed from C57BL/6ByJ and BALBc/ByJ identified strains that showed either no change, a decrease, or an increase in the EP acutely after noise (Ohlemiller et al., 2016). Underlying all this may be fundamentally different stress responses in the cochlear lateral wall in B6 vs. BALB, CBA/J, and CBA/CaJ mice (Ohlemiller and Gagnon, 2007; Ohlemiller et al., 2011; Herranen et al., 2018). If such basic properties can vary within species, then we may certainly expect further discrepancies among mice, guinea pigs, gerbils, chinchillas, and humans.

A common observation is for very intense exposures to promote edema of the intrastrial space (e.g., Duvall et al., 1974; Úlehlová, 1983; Duvall and Robinson, 1987). The swelling typically resolves within a few days, by which time the EP generally returns to normal. Such severe exposures usually can be shown to open the reticular lamina, allowing uncontrolled mixing of endolymph and perilymph (Hirose and Liberman, 2003; Hirose et al., 2005). In such cases, acute changes in the EP can be largely attributed to this breach of the lamina, yet even in these cases the EP often normalizes. To date, there is little evidence of noise causing direct tears in strial capillaries or in the stria itself. The likely cause of strial swelling, though not proven, is osmotic imbalance of the intrastrial fluids caused by metabolic exhaustion of the strial marginal cells. Although there appear to be no published data, intense noise may greatly increase the amount of K+ being shuttled back to the stria by the spiral ligament and released into the intrastrial space, placing pressure on marginal cells to remove it. Noise exposures that massively damage the organ of Corti and breach the integrity of the reticular lamina tell us little about nuances of strial capillary transport because they are dwarfed by other changes. Moreover, a stria that is attempting to generate an EP in a scala media riddled with holes will likely show exacerbated damage. In summary, there is consensus that noise exposure can damage strial capillaries, but little clear evidence that altered strial capillary transport by any mechanism can permanently lower the EP, or is a significant driver of noise-induced hearing loss.

## Strial barrier dysfunction in ototoxicity

Commonly discussed ototoxins include loop diuretics (e.g., ethacrynic acid, furosemide), aminoglycoside antibiotics (e.g., kanamycin, gentamicin), and anti-neoplastics (e.g., cisplatin). Each of these appear to pass readily from strial capillaries into the intrastrial space, and the transfer of aminoglycosides into scala media by marginal cells has been elegantly worked out by Steyger et al. (Dai and Steyger, 2008; Li and Steyger, 2011). Loop diuretics and cisplatin can reversibly or permanently damage the stria and EP, albeit for different reasons. Furosemide interferes with the Na+/K+/Cl- co-transporter in strial marginal cells, causing intrastrial K+ and Na+ to rise along with the osmolarity of the intrastrial fluid, thus drawing water and triggering edema (Naito and Watanabe, 1997; Azuma et al., 2002). Furosemide has proven helpful in promoting the access of aminoglycosides to cochlear fluid spaces, thereby exacerbating experimental hair cell lesions (Hirose and Sato, 2011). The mechanism may be increased transcellular trafficking by marginal cells (Naito and Watanabe, 1997). Cisplatin is broadly toxic to cells due to its effects on DNA, and within the cochlea, directly damages hair cells, neurons, and the stria (Meech et al., 1998; Yu et al., 2022). Some studies (Zhang et al., 2020a; Gu et al., 2022) have claimed a role for paracellular strial capillary leak in hearing loss after cisplatin application in animals, yet without demonstrating any causal link (Laurell et al., 1997). Although aminoglycosides can exert primary effects on stria (Forge and Fradis, 1985; Forge et al., 1987), these take the form of thinning and modest cell loss, and EP reduction is not typically invoked as a cause of hearing loss following aminoglycoside application. In sum, while some ototoxins may directly affect the stria and EP, we find no compelling claims that strial barrier function *per se* is a significant factor.

## Strial barrier dysfunction in autoimmune disease

Human autoimmune diseases often have a hearing loss component, and temporal bone studies of autoimmune disease subjects consistently demonstrate stria vascularis and organ of Corti pathology (Trune, 2002). The major autoimmune diseases include lupus erythematosus, type I diabetes, and Sjogren's disease, all of which involve inflammatory infiltration and destruction of organs and connective tissue. The most common finding among mouse autoimmune models, including C3H-Fas^lpr^, MRL-Fas^lpr^, and Palmerston North is strial dysfunction (Trune et al., 1989; Ruckenstein et al., 1999a,b; Trune, 2002). The best understood autoimmune models have involved the Fas-Fas Ligand signaling system, which induces inflammatory cell death but also mediates proliferative and activating signals (Wajant, 2002). The *lpr* allele of the Fas locus (lymphoproliferation, also known as APO-1, CD95, TNFR6, and Tnfrsf6) is an autosomal recessive mutation causing lymphoproliferation and autoimmunity in mice. A remarkable feature of the mouse autoimmune models is that the stria does not degenerate, even though there may be EP reduction. In fact, the marked loss of strial volume in MRL-Fas^lpr^ mice can be largely reversed by application of steroids (Trune and Kempton, 2001), suggesting that the loss of strial volume in these mice occurs through retraction of cell processes, not cell loss. Among other features of both Fas^lpr^ models is that IgG extravasates and binds to endothelial cells and capillary basement membranes (Trune, 1997; Ruckenstein and Hu, 1999), but we showed in Figure 2 that this can occur normally in some models. Older *lpr* mutants may also exhibit strial edema and striking thickness changes in capillary basement membranes (Schwartz et al., 1992). It remains unclear why some mouse autoimmune models show hearing loss. EP reduction has only been directly demonstrated for one model (Ruckenstein et al., 1999b). The final point here is that a recent attempt to confirm Ruckenstein et al.s' results in the same mouse (MRL-Fas^lpr^) failed to identify the published phenotype in the current commercial model (Mills et al., in prep), suggesting that the line has been lost.

Other conditions that may involve vascular pathology and potentially immune-mediated disease include endolymphatic hydrops, Meniere's disease, sudden sensorineural hearing loss (SSNHL), and Alport Syndrome. Meniere's disease and SSNHL and have been reviewed recently (Yu et al., 2021; Johns et al., 2022; Thulasiram et al., 2022; Shi, 2023), describing a common finding of strial degeneration. Alport syndrome is a condition associated with genetic variation in type IV collagen (Lin and Trune, 1997). Alport's syndrome results in renal insufficiency and renal failure and progressive sensorineural hearing loss associated with progressive thickening of strial capillary basement membranes and the dysregulation of extracellular matrix proteins (Thulasiram et al., 2022). Mouse models of Alport's syndrome created by a inserting the human collagen variant that causes Alport's in humans, did not result in hearing loss in mice (Cosgrove et al., 1998) or show the same anatomic features (Johnsson and Arenberg, 1981; Merchant et al., 2004), which range from subtle anomalies of basement membranes to strial degeneration. Some have reported that Alport mice on a 129/Sv background are more vulnerable to noise, which is asserted to be a reflection of thickened strial capillary basement membranes and resulting hypoperfusion (Meehan et al., 2016; Dufek et al., 2020). In any case, across this literature there is no theme of abnormal strial capillary transport as a cause of hearing loss.

## Strial barrier dysfunction in induced inflammation

Inflammation has been implicated in strial dysfunction, and a number of studies have modeled systemic or local inflammation in mice by applying lipopolysaccharide (LPS, or endotoxin). Application of LPS to the middle ears of guinea pigs resulted in HRP accumulation in the intrastrial space (Watanabe and Tanaka, 1997), and also caused degenerative changes (Watanabe et al., 2001). In separate studies in B6 and BALB mice (Zhang et al., 2015; Jiang et al., 2019), LPS injected into the middle ear caused hearing loss, but this could have had a middle ear component, and no EP recordings or histopathology were carried out to examine any cochlear basis. *In vivo* imaging indicated FITC-dextran (70 kDa) or FITC-albumin extravasating from the lumen of strial capillaries, and strial capillary endothelial cells were shown to have irregular luminal surfaces, suggestive of distorted tight junctions. Additional analysis of strial capillaries indicated a loss of tight junction proteins ZO-1 and occludin. LPS is known to cause capillary leakage from vascular beds in the lung and the liver, and this effect is replicated in the inner ear. However, two studies involving intraperitoneal injection of LPS into B6 mice reported no threshold shift or significant EP reduction over a 5-day observation, despite clear recruitment of macrophages into the spiral ligament and other indications of inflammation (Hirose et al., 2014b; Hirose and Li, 2019). It is difficult to reconcile these very different mouse results, except for the mode of LPS application. Intratympanic LPS (Zhang et al., 2015; Jiang et al., 2019), may promote more direct hair cell and neural effects, in keeping with reported cochleotoxic effects of otitis media (e.g., Guo et al., 1994; da Costa Monsanto et al., 2017). The stronger tendency toward degenerative changes in the stria of guinea pigs may indicate a species difference. Nevertheless, in sum, there is no consistent compelling evidence that endotoxins promote hearing loss primarily by altering strial capillary barrier function.

## Strial barrier dysfunction in genetic hearing loss

Mouse transgenic and knockout models have been used effectively and extensively to probe strial function. Much of our knowledge of the critical strial components for endolymph and EP generation are based on knockout models for claudin 11 (Gow et al., 2004), Kir4.1 (Marcus et al., 2002), the Na+/K+/Cl- co-transporter (Delpire et al., 1999), and others. Many genes principally impacting strial function are known deafness genes, including those governing Norrie disease, Alport syndrome, Meniere's disease, Waardenburg syndrome, and Pendred syndrome (Yu et al., 2021; Thulasiram et al., 2022). In none of these conditions, however, has it been necessary to invoke impaired strial barrier function as a primary driver of disease. The most prominently cited paper in this regard involved a mouse KO for connexin 30 (Cx30) (Cohen-Salmon et al., 2007; see also Chen et al., 2014, 2022), in which strial capillary leak was implicated in EP reduction and hearing loss. But subsequent studies revealed that mice were actually connexin 26/30 double KOs with broader degeneration, and that delimited Cx30 KOs did not show hearing loss or EP reduction (Boulay et al., 2013). Down-regulation of Connexin 43 (Cx43) using siRNA (Zhang et al., 2020b) was also reported to cause EP reduction and hearing loss in B6 mice. The purported mechanism for this was loss of strial capillary barrier integrity owing to the role of Cx43 in forming tight junctions, and a loss of barrier integrity in cultured endothelial cells provided support for such a mechanism. However, Cx43 is expressed in a wide array of tight junctions (Strauss and Gourdie, 2020), and has been identified in the organ of Corti and in strial basal cells (Suzuki et al., 2003; Liu et al., 2011). In either location it could be required for critical barrier integrity in a manner having nothing to do with strial capillaries. The authors also suggested that Cx43 helps mediate K+ transfer from the spiral ligament to strial basal cells, and ultimately the intrastrial space. Such a role could lower the EP by interfering with the K+ supply to marginal cells. Again, however, this would have little to do with strial capillary integrity. Finally, as mentioned earlier, genetic ablation of pericytes or PVMs (Zhang et al., 2012, 2021; Shi, 2023) does not consistently support strial barrier dysfunction as a primary cause of hearing loss.

## Conclusions

The stria vascularis performs multiple functions that are vital for hearing. Therefore, compromise of this structure by genetic or environmental damage to any of its critical channels, pumps, or transporters will also compromise hearing. Because of the stria's high metabolic rate, altered strial capillary function could also adversely affect the endocochlear potential and hearing thresholds. This review has explored the notion that impaired strial capillary barrier function alone can compromise strial function, the EP, and hearing. This has been suggested for a number of conditions, including aging, noise, autoimmune disease, and inflammation. Instead, we find that strial capillaries are not particularly exclusive in their transport of small molecules and proteins, and that this occurs in a number of animal models and conditions. The most exclusive barrier in the stria appears to be the marginal cells, which solely determine what passes from the stria to the endolymph. Moreover, essential properties of strial capillary transport, such as what transport mechanism facilitates the passage of what type of molecule, vary across and within species. This argues against any single feature of strial capillary transport as being absolutely required for a normal EP and sensitive hearing. Overall, we find little evidence that strial capillary leakage, by whatever mechanism, can impair EP generation or hearing. By extension, strial capillary leakage is not a primary driver of hearing loss, nor a significant sequela of other conditions, and does not merit targeted therapies. The surprisingly free transfer of metabolites between the capillary lumen and intrastrial space may reflect adaptive processes that we do not fully understand, and some general concepts that apply to other vascular barriers may not apply to this vascular bed.

## Author contributions

KO: Conceptualization, Data curation, Formal analysis, Investigation, Methodology, Project administration, Resources, Supervision, Visualization, Writing – original draft, Writing – review & editing. ND: Data curation, Formal analysis, Investigation, Writing – review & editing. VH: Data curation, Formal analysis, Investigation, Writing – review & editing. KF: Data curation, Formal analysis, Investigation, Writing – review & editing. KH: Conceptualization, Writing – original draft, Writing – review & editing.

## References

[B1] Ando M. Edamatsu M. Fukuizumi S. Takeuchi S. (2008). Cellular localization of facilitated glucose transporter 1 (GLUT-1) in the cochlear stria vascularis: its possible contribution to the transcellular glucose pathway. Cell Tissue Res. 331, 763–769. 10.1007/s00441-007-0495-2 18196278

[B2] Armstrong S. M. Khajoee V. Wang C. Wang T. Tigdi J. Yin J.. (2012). Co-regulation of transcellular and paracellular leak across microvascular endothelium by dynamin and Rac. Am. J. Pathol. 180, 1308–1323. 10.1016/j.ajpath.2011.12.002 22203054

[B3] Armulik A. Abramsson A. Betsholtz C. (2005). Endothelial/pericyte interactions. Circ. Res. 97, 512–523. 10.1161/01.RES.0000182903.16652.d7 16166562

[B4] Attwell D. Mishra A. Hall C. N. O'Farrell F. M. Dalkara T. (2016). What is a pericyte? J. Cereb. Blood Flow Metab. 36, 451–455. 10.1177/0271678X15610340 26661200 PMC4759679

[B5] Axelsson A. (1988). Comparative anatomy of cochlear blood vessels. Am. J. Otolaryngol. 9, 278–290. 10.1016/S0196-0709(88)80036-X 3067591

[B6] Azuma H. Takeuchi S. Higashiyama K. Ando M. Kakigi A. Nakahira M.. (2002). Bumetanide-induced enlargement of the intercellular space in the stria vascularis requires an active Na+-K+-ATPase. Acta Otolaryngol. 122, 816–821. 10.1080/003655402_000028051 12542198

[B7] Bae S. H. Yoo J. E. Choe Y. H. Kwak S. H. Choi J. Y. Jung J.. (2021). Neutrophils infiltrate into the spiral ligament but not the stria vascularis in the cochlea during lipopolysaccharide-induced inflammation. Theranostics 11:2522. 10.7150/thno.49121 33456557 PMC7806478

[B8] Boulay A. C. Del Castillo F. J. Giraudet F. Hamard G. Giaume C. Petit C.. (2013). Hearing is normal without Connexin 30. J. Neurosci. 33, 430–434. 10.1523/JNEUROSCI.4240-12.2013 23303923 PMC6704917

[B9] Boussaty E. C. Tedeschi N. Novotny M. Ninoyu Y. Du E. Draf C.. (2023). Cochlear transcriptome analysis of an outbred mouse population (CFW). bioRxiv. 10.3389/fncel.2023.1256619 38094513 PMC10716316

[B10] Cable J. Steel K. P. (1991). Identification of two types of melanocyte within the stria vascularis of the mouse inner ear. Pigment Cell Res. 4, 87–101. 10.1111/j.1600-0749.1991.tb00320.x 1946214

[B11] Chang Q. Tang W. Ahmad S. Zhou B. Lin X. (2008). Gap junction mediated intercellular metabolite transfer in the cochlea is compromised in Connexin 30 null mice. PLoS ONE 3:e4088. 10.1371/journal.pone.0004088 19116647 PMC2605248

[B12] Chen J. Chen J. Zhu Y. Liang C. Zhao H. B. (2014). Deafness induced by Connexin 26 (GJB2) deficiency is not determined by endocochlear potential (EP) reduction but is associated with cochlear developmental disorders. Biochem. Biophys. Res. Commun. 448, 28–32. 10.1016/j.bbrc.2014.04.016 24732355 PMC4105360

[B13] Chen J. Chen P. He B. Gong T. Li Y. Zhang J.. (2022). Connexin30-deficiency causes mild hearing loss with the reduction of endocochlear potential and ATP release. Front. Cell. Neurosci. 15:819194. 10.3389/fncel.2021.819194 35110999 PMC8802669

[B14] Coffin A. B. Boney R. Hill J. Tian C. Steyger P. S. (2021). Detecting novel ototoxins and potentiation of ototoxicity by disease settings. Front. Neurol. 12:725566. 10.3389/fneur.2021.725566 34489859 PMC8418111

[B15] Cohen-Salmon M. Regnault B. Cayet N. Caille D. Demuth K. Hardelin J. P.. (2007). Connexin30 deficiency causes instrastrial fluid–blood barrier disruption within the cochlear stria vascularis. Proc. Nat. Acad. Sci. U. S. A. 104, 6229–6234. 10.1073/pnas.0605108104 17400755 PMC1851033

[B16] Conlee J. W. Gerity L. C. Bennett M. L. (1994). Ongoing proliferation of melanocytes in the stria vascularis of adult guinea pigs. Hear. Res. 79, 115–122. 10.1016/0378-5955(94)90133-3 7806474

[B17] Conlee J. W. Parks T. N. Schwartz I. R. Creel D. J. (1989). Comparative anatomy of melanin pigment in the stria vascularis: evidence for a distinction between melanocytes and intermediate cells in the cat. Acta Otolaryngol. 107, 48–58. 10.3109/00016488909127478 2929316

[B18] Cosgrove D. Samuelson G. Meehan D. T. Miller C. McGee J. Walsh E. J.. (1998). Ultrastructural, physiological, and molecular defects in the inner ear of a gene-knockout mouse model for autosomal Alport syndrome. Hear. Res. 121, 84–98. 10.1016/S0378-5955(98)00069-0 9682811

[B19] da Costa Monsanto R. Schachern P. Paparella M. M. Cureoglu S. de Oliveira Penido N. (2017). Progression of changes in the sensorial elements of the cochlear and peripheral vestibular systems: the otitis media continuum. Hear. Res. 351, 2–10. 10.1016/j.heares.2017.05.003 28578877 PMC6557455

[B20] Dai C. F. Steyger P. S. (2008). A systemic gentamicin pathway across the stria vascularis. Hear. Res. 235, 114–124. 10.1016/j.heares.2007.10.010 18082985 PMC2703593

[B21] Daneman R. Zhou L. Kebede A. A. Barres B. A. (2010). Pericytes are required for blood–brain barrier integrity during embryogenesis. Nature 468, 562–566. 10.1038/nature09513 20944625 PMC3241506

[B22] Delpire E. Lu J. England R. Dull C. Thorne T. (1999). Deafness and imbalance associated with inactivation of the secretory Na-K-2Cl co-transporter. Nat. Genet. 22, 192–195. 10.1038/9713 10369265

[B23] Dufek B. Meehan D. T. Delimont D. Samuelson G. Madison J. Shi X.. (2020). Pericyte abnormalities precede strial capillary basement membrane thickening in Alport mice. Hear. Res. 390:107935. 10.1016/j.heares.2020.107935 32234583 PMC7491280

[B24] Duvall A. J. Quick C. A. Sutherland C. R. (1971). Horseradish peroxidase in the lateral cochlear wall: an electron microscopic study of transport. Arch. Otolaryngol. 93, 304–316. 10.1001/archotol.1971.00770060442015 5542356

[B25] Duvall A. J. Robinson K. S. (1987). Local vs systemic effects of acoustic trauma on cochlear structure and transport. Arch. Otolaryngol. Head Neck Surg. 113, 1066–1071. 10.1001/archotol.1987.01860100044019 3620127

[B26] Duvall A. J. III. Hukee M. J. Santi P. A. (1979). The morphologic effects of histamine on the lateral cochlear wall. Otolaryngol. Head Neck Surg. 87, 666–684. 10.1177/019459987908700523 503533

[B27] Duvall A. J. III. Klinkner A. (1983). Macromolecular tracers in the mammalian cochlea. Am. J. Otolaryngol. 4, 400–410. 10.1016/S0196-0709(83)80046-5 6660366

[B28] Duvall A. J. III. Ward W. D. Lauhala K. E. (1974). Stria ultrastructure and vessel transport in acoustic trauma. Ann. Otol. Rhinol. Laryngol. 83, 498–514. 10.1177/000348947408300413 4859492

[B29] Dwyer N. Y. (2010). “Strial capillary permeability following noise exposure in mice. Independent Studies and Capstones,” in Paper 611. Program in Audiology and Communication Sciences (Washington University School of Medicine). Available online at: https://digitalcommons.wustl.edu/pacs_capstones/611 (accessed December, 2023).

[B30] Fahrenthold K. C. (2015). “Strial capillary permeability studied with fluorescent tracers in inbred mice,” in Independent Studies and Capstones. Paper 714. Program in Audiology and Communication Sciences (Washington University School of Medicine). Available online at: https://digitalcommons.wustl.edu/pacs_capstones/714 (accessed December, 2023).

[B31] Ferrary E. Sterkers O. Saumon G. Tran Ba Huy P. Amiel C. (1987). Facilitated transfer of glucose from blood into perilymph in the rat cochlea. Am. J. Physiol. Renal Physiol. 253, F59–F65. 10.1152/ajprenal.1987.253.1.F59 3111276

[B32] Filipo R. Barbara M. Cordier A. Mafera B. Romeo R. Attanasio G.. (1997). Osmotic drugs in the treatment of cochlear disorders: a clinical and experimental study. Acta Otolaryngol. 117, 229–231. 10.3109/00016489709117777 9105456

[B33] Firbas W. Gruber H. Wicke W. (1981). The blood vessels of the limbus spiralis. Arch. Otorhinolaryngol. 232, 131–137. 10.1007/BF00505032 7271586

[B34] Floc'h J. L. Tan W. Telang R. S. Vlajkovic S. M. Nuttall A. Rooney W. D.. (2014). Markers of cochlear inflammation using MRI. J. Magn. Reson. Imaging 39, 150–161. 10.1002/jmri.24144 23589173 PMC3935384

[B35] Forge A. Fradis M. (1985). Structural abnormalities in the stria vascularis following chronic gentamicin treatment. Hear. Res. 20, 233–244. 10.1016/0378-5955(85)90028-0 4086385

[B36] Forge A. Wright A. Davies S. J. (1987). Analysis of structural changes in the stria vascularis following chronic gentamicin treatment. Hear. Res. 31, 253–265. 10.1016/0378-5955(87)90195-X 3436852

[B37] Geevarghese A. Herman I. M. (2014). Pericyte-endothelial crosstalk: implications and opportunities for advanced cellular therapies. Transl. Res. 163, 296–306. 10.1016/j.trsl.2014.01.011 24530608 PMC3976718

[B38] Gleich O. Piña A. L. (2008). Protein expression of pigment-epithelium-derived factor in rat cochlea. Cell Tissue Res. 332, 565–571. 10.1007/s00441-008-0608-6 18418629

[B39] Goldwyn B. G. Quirk W. S. (1997). Calcium channel blockade reduces noise-induced vascular permeability in cochlear stria vascularis. Laryngoscope 107, 1112–1116. 10.1097/00005537-199708000-00019 9261017

[B40] Gorgas K. Jahnke K. (1974). The permeability of blood vessels in the guinea pig cochlea: II. Vessels in the spiral ligament and the stria vascularis. Brain Struct. Funct. 146, 33–42. 10.1007/BF00341381 4618724

[B41] Gow A. Davies C. Southwood C. M. Frolenkov G. Chrustowski M. Ng L.. (2004). Deafness in Claudin 11-null mice reveals the critical contribution of basal cell tight junctions to stria vascularis function. J. Neurosci. 24, 7051–7062. 10.1523/JNEUROSCI.1640-04.2004 15306639 PMC4615685

[B42] Gratton M. A. Rao V. H. Meehan D. T. Askew C. Cosgrove D. (2005). Matrix metalloproteinase dysregulation in the stria vascularis of mice with Alport syndrome: implications for capillary basement membrane pathology. Am. J. Pathol. 166, 1465–1474. 10.1016/S0002-9440(10)62363-2 15855646 PMC1606400

[B43] Gratton M. A. Schmiedt R. A. Schulte B. A. (1996). Age-related decreases in endocochlear potential are associated with vascular abnormalities in the stria vascularis. Hear. Res. 102, 181–190. 10.1016/S0378-5955(96)90017-9 8951461

[B44] Gratton M. A. Schulte B. A. Smythe N. M. (1997). Quantification of the stria vascularis and strial capillary areas in quiet-reared young and aged gerbils. Hear. Res. 114, 1–9. 10.1016/S0378-5955(97)00025-7 9447913

[B45] Gratton M. A. Smyth B. J. Schulte B. A. Vincent Jr D. A. (1995). Na, K-ATPase activity decreases in the cochlear lateral wall of quiet-aged gerbils. Hear. Res. 83, 43–50. 10.1016/0378-5955(94)00188-V 7607990

[B46] Groothuis D. R. Vavra M. W. Schlageter K. E. Kang E. W. Itskovich A. C. Hertzler S.. (2007). Efflux of drugs and solutes from brain: the interactive roles of diffusional transcapillary transport, bulk flow and capillary transporters. J. Cereb. Blood Flow Metab. 27, 43–56. 10.1038/sj.jcbfm.9600315 16639426

[B47] Gu J. Tong L. Lin X. Chen Y. Wu H. Wang X.. (2022). The disruption and hyperpermeability of blood-labyrinth barrier mediates cisplatin-induced ototoxicity. Toxicol. Lett. 354, 56–64. 10.1016/j.toxlet.2021.10.015 34757176

[B48] Guo Y. Wu Y. Chen W. Lin J. (1994). Endotoxic damage to the stria vascularis: the pathogenesis of sensorineural hearing loss secondary to otitis media? J. Laryngol. Otol. 108, 310–313. 10.1017/S0022215100126623 8182316

[B49] Hayashi H. Sone M. Schachern P. A. Wakamatsu K. Paparella M. M. Nakashima T. (2007). Comparison of the quantity of cochlear melanin in young and old C57BL/6 mice. Arch. Otolaryngol. Head Neck Surg. 133, 151–154. 10.1001/archotol.133.2.151 17309984

[B50] He P. Curry F. E. (1995). Measurement of membrane potential of endothelial cells in single perfused microvessels. Microvasc. Res. 50, 183–198. 10.1006/mvre.1995.1052 8538499

[B51] Henson V. E. (2013). “Normal cochlear lateral wall permeability to fluorescent macromolecules,” in Independent Studies and Capstones. Paper 660. Program in Audiology and Communication Sciences (Washington University School of Medicine). Available online at: https://digitalcommons.wustl.edu/pacs_capstones/660 (accessed December, 2023).

[B52] Herranen A. Ikäheimo K. Virkkala J. Pirvola U. (2018). The stress response in the non-sensory cells of the cochlea under pathological conditions—possible role in mediating noise vulnerability. J. Assoc. Res. Otolaryngol. 19, 637–652. 10.1007/s10162-018-00691-2 30191426 PMC6249157

[B53] Hirose K. Discolo C. M. Keasler J. R. Ransohoff R. (2005). Mononuclear phagocytes migrate into the murine cochlea after acoustic trauma. J. Comp. Neurol. 489, 180–194. 10.1002/cne.20619 15983998

[B54] Hirose K. Hartsock J. J. Johnson S. Santi P. Salt A. N. (2014a). Systemic lipopolysaccharide compromises the blood-labyrinth barrier and increases entry of serum fluorescein into the perilymph. J. Assoc. Res. Otolaryngol. 15, 707–719. 10.1007/s10162-014-0476-6 24952083 PMC4164684

[B55] Hirose K. Li S. Z. (2019). The role of monocytes and macrophages in the dynamic permeability of the blood-perilymph barrier. Hear. Res. 374, 49–57. 10.1016/j.heares.2019.01.006 30710792 PMC6459018

[B56] Hirose K. Li S. Z. Ohlemiller K. K. Ransohoff R. M. (2014b). Systemic lipopolysaccharide induces cochlear inflammation and exacerbates the synergistic ototoxicity of kanamycin and furosemide. J. Assoc. Res. Otolaryngol. 15, 555–570. 10.1007/s10162-014-0458-8 24845404 PMC4141430

[B57] Hirose K. Liberman M. C. (2003). Lateral wall histopathology and endocochlear potential in the noise-damaged mouse cochlea. J. Assoc. Res. Otolaryngol. 4, 339–352. 10.1007/s10162-002-3036-4 14690052 PMC1805786

[B58] Hirose K. Sato E. (2011). Comparative analysis of combination kanamycin-furosemide versus kanamycin alone in the mouse cochlea. Hear. Res. 272, 108–116. 10.1016/j.heares.2010.10.011 21044672 PMC4519356

[B59] Hishikawa S. Edamatsu M. Inoue-Ikeda R. Ando M. (2015). Direct evidence of the glucose uptake into cochlear strial marginal cells: application of a fluorescent tracer method combined with immunohistochemistry. Bioimages 23, 1–8.

[B60] Hosokawa S. Hosokawa K. Ishiyama G. Ishiyama A. Lopez I. A. (2018). Immunohistochemical localization of megalin and cubilin in the human inner ear. Brain Res. 1701, 153–160. 10.1016/j.brainres.2018.09.016 30218661 PMC6289768

[B61] Hou Z. Neng L. Zhang J. Cai J. Wang X. Zhang Y.. (2020). Acoustic trauma causes cochlear pericyte-to-myofibroblast–like cell transformation and vascular degeneration, and transplantation of new pericytes prevents vascular atrophy. Am. J. Pathol. 190, 1943–1959. 10.1016/j.ajpath.2020.05.019 32562655 PMC7450261

[B62] Hou Z. Wang X. Cai J. Zhang J. Hassan A. Auer M.. (2018). Platelet-derived growth factor subunit B signaling promotes pericyte migration in response to loud sound in the cochlear stria vascularis. J. Assoc. Res. Otolaryngol. 19, 363–379. 10.1007/s10162-018-0670-z 29869048 PMC6081892

[B63] Hukee M. J. Duvall A. J. III. (1985). Cochlear vessel permeability to horseradish peroxidase in the normal and acoustically traumatized chinchilla: a reevaluation. Ann. Otol. Rhinol. Laryngol. 94, 297–303. 10.1177/000348948509400316 4014952

[B64] Ito T. Kurata N. Fukunaga Y. (2022). Tissue-resident macrophages in the Stria Vascularis. Front. Neurol. 13:818395. 10.3389/fneur.2022.818395 35185769 PMC8850293

[B65] Jiang W. J. Zhou Z. Wang Y. P. Gao W. Li L. Si J. Q. (2023). PGC-1α affects cochlear pericytes migration in noise-exposed mice. Biochem. Biophys. Res. Commun. 687:149172. 10.1016/j.bbrc.2023.149172 37931421

[B66] Jiang Y. Zhang J. Rao Y. Chen J. Chen K. Tang Y. (2019). Lipopolysaccharide disrupts the cochlear blood-labyrinth barrier by activating perivascular resident macrophages and up-regulating MMP-9. Int. J. Pediatr. Otorhinolaryngol. 127:109656. 10.1016/j.ijporl.2019.109656 31470202

[B67] Johns J. D. Adadey S. M. Hoa M. H. (2022). The role of the stria vascularis in neglected otologic disease. Hear. Res. 428:108682. 10.1016/j.heares.2022.108682 36584545 PMC9840708

[B68] Johnsson L. G. Arenberg I. K. (1981). Cochlear abnormalities in Alport's syndrome. Arch. Otolaryngol. 107, 340–349. 10.1001/archotol.1981.00790420014004 7224962

[B69] Keithley E. M. (2022). Inner ear immunity. Hear. Res. 419:108518. 10.1016/j.heares.2022.108518 35584985

[B70] Kim J. Ricci A. J. (2022). *In vivo* real-time imaging reveals megalin as the aminoglycoside gentamicin transporter into cochlea whose inhibition is otoprotective. Proc. Nat. Acad. Sci. U. S. A. 119:e2117946119. 10.1073/pnas.2117946119 35197290 PMC8892513

[B71] Kottke M. A. Walters T. J. (2016). Where's the leak in vascular barriers? A review. Shock 46, 20–36. 10.1097/SHK.0000000000000666 27405062

[B72] Laurell G. Teixeira M. Sterkers O. Ferrary E. (1997). Paracellular transport properties of inner ear barriers do not account for cisplatin toxicity in the rat. Hear. Res. 110, 135–140. 10.1016/S0378-5955(97)00067-1 9282895

[B73] Lawrence M. (1974). Direct visualization of living organ of corti and studies of its extracellular fluids. Laryngoscope 84, 1767–1776. 10.1288/00005537-197410000-00011 4418561

[B74] Le T. N. Blakley B. W. (2017). Mannitol and the blood-labyrinth barrier. J. Otolaryngol. Head Neck Surg. 46, 1–7. 10.1186/s40463-017-0245-8 29228990 PMC5725891

[B75] Li H. Steyger P. S. (2011). Systemic aminoglycosides are trafficked via endolymph into cochlear hair cells. Sci. Rep. 1:159. 10.1038/srep00159 22355674 PMC3240991

[B76] Lin D. W. Trune D. R. (1997). Breakdown of stria vascularis blood-labyrinth barrier in C3H/lpr autoimmune disease mice. Otolaryngol. Head Neck Surg. 117, 530–534. 10.1016/S0194-5998(97)70026-3 9374179

[B77] Liu W. Boström M. Kinnefors A. Edin F. Rask-Andersen H. (2011). Connexin 43 expression in the human cochlea: an immunohistochemistry study. J. Hear. Sci. 1, 21–29. 10.17430/882155

[B78] Marcus D. C. Wu T. Wangemann P. Kofuji P. (2002). KCNJ10 (Kir4. 1) potassium channel knockout abolishes endocochlear potential. Am. J. Physiol. Cell Physiol. 282, C403–C407. 10.1152/ajpcell.00312.2001 11788352

[B79] Matschinsky F. M. Thalmann R. (1967). L quantitative histochemistry of microscopic structures of the cochlea: II. Ischemic alterations of levels of glycolytic intermediates and cofactors in the organ of corti and stria vascularis. Ann. Otol. Rhinol. Laryngol. 76, 638–646. 10.1177/000348946707600309 4382918

[B80] Meech R. P. Campbell K. C. Hughes L. P. Rybak L. P. (1998). A semiquantitative analysis of the effects of cisplatin on the rat stria vascularis. Hear. Res. 124, 44–59. 10.1016/S0378-5955(98)00116-6 9822901

[B81] Meehan D. T. Delimont D. Dufek B. Zallocchi M. Phillips G. Gratton M. A.. (2016). Endothelin-1 mediated induction of extracellular matrix genes in strial marginal cells underlies strial pathology in Alport mice. Hear. Res. 341, 100–108. 10.1016/j.heares.2016.08.003 27553900 PMC5086449

[B82] Merchant S. N. Burgess B. J. Adams J. C. Kashtan C. E. Gregory M. C. Santi P. A.. (2004). Temporal bone histopathology in alport syndrome. Laryngoscope 114, 1609–1618. 10.1097/00005537-200409000-00020 15475791

[B83] Muñoz D. J. Kendrick I. S. Rassam M. Thorne P. R. (2001). Vesicular storage of adenosine triphosphate in the guinea-pig cochlear lateral wall and concentrations of ATP in the endolymph during sound exposure and hypoxia. Acta Otolaryngol. 121, 10–15. 10.1080/000164801300006209 11270486

[B84] Muradashvili N. Tyagi R. Lominadze D. (2012). A dual-tracer method for differentiating transendothelial transport from paracellular leakage *in vivo* and *in vitro*. Front. Physiol. 3:166. 10.3389/fphys.2012.00166 22754530 PMC3385581

[B85] Naito H. Watanabe K. (1997). Alteration in capillary permeability of horseradish peroxidase in the stria vascularis and movement of leaked horseradish peroxidase after administration of furosemide. ORL 59, 248–257. 10.1159/000276948 9279862

[B86] Neng L. Zhang F. Kachelmeier A. Shi X. (2013). Endothelial cell, pericyte, and perivascular resident macrophage-type melanocyte interactions regulate cochlear intrastrial fluid–blood barrier permeability. J. Assoc. Res. Otolaryngol. 14, 175–185. 10.1007/s10162-012-0365-9 23247886 PMC3660918

[B87] Neng L. Zhang J. Yang J. Zhang F. Lopez I. A. Dong M.. (2015). Structural changes in the strial blood–labyrinth barrier of aged C57BL/6 mice. Cell Tissue Res. 361, 685–696. 10.1007/s00441-015-2147-2 25740201 PMC4552584

[B88] Nin F. Hibino H. Doi K. Suzuki T. Hisa Y. Kurachi Y. (2008). The endocochlear potential depends on two K+ diffusion potentials and an electrical barrier in the stria vascularis of the inner ear. Proc. Nat. Acad. Sci. U. S. A. 105, 1751–1756. 10.1073/pnas.0711463105 18218777 PMC2234216

[B89] Noble K. Brown L. Elvis P. Lang H. (2021). Cochlear immune response in presbyacusis: a focus on dysregulation of macrophage activity. J. Assoc. Res. Otolaryngol. 23, 1–16. 10.1007/s10162-021-00819-x 34642854 PMC8782976

[B90] Ohlemiller K. K. (2009). Mechanisms and genes in human strial presbycusis from animal models. Brain Res. 1277, 70–83. 10.1016/j.brainres.2009.02.079 19285967 PMC2792931

[B91] Ohlemiller K. K. Dahl A. R. Gagnon P. M. (2010). Divergent aging characteristics in CBA/J and CBA/CaJ mouse cochleae. J. Assoc. Res. Otolaryngol. 11, 605–623. 10.1007/s10162-010-0228-1 20706857 PMC2975886

[B92] Ohlemiller K. K. Gagnon P. M. (2007). Genetic dependence of cochlear cells and structures injured by noise. Hear. Res. 224, 34–50. 10.1016/j.heares.2006.11.005 17175124 PMC1809471

[B93] Ohlemiller K. K. Kaur T. Warchol M. E. Withnell R. H. (2018). The endocochlear potential as an indicator of reticular lamina integrity after noise exposure in mice. Hear. Res. 361, 138–151. 10.1016/j.heares.2018.01.015 29426600 PMC5967872

[B94] Ohlemiller K. K. Kiener A. L. Gagnon P. M. (2016). QTL mapping of endocochlear potential differences between C57BL/6J and BALB/cJ mice. J. Assoc. Res. Otolaryngol. 17, 173–194. 10.1007/s10162-016-0558-8 26980469 PMC4854825

[B95] Ohlemiller K. K. Lett J. M. Gagnon P. M. (2006). Cellular correlates of age-related endocochlear potential reduction in a mouse model. Hear. Res. 220, 10–26. 10.1016/j.heares.2006.06.012 16901664

[B96] Ohlemiller K. K. Rice M. E. R. Lett J. M. Gagnon P. M. (2009). Absence of strial melanin coincides with age-associated marginal cell loss and endocochlear potential decline. Hear. Res. 249, 1–14. 10.1016/j.heares.2008.12.005 19141317

[B97] Ohlemiller K. K. Rosen A. D. Rellinger E. A. Montgomery S. C. Gagnon P. M. (2011). Different cellular and genetic basis of noise-related endocochlear potential reduction in CBA/J and BALB/cJ mice. J. Assoc. Res. Otolaryngol. 12, 45–58. 10.1007/s10162-010-0238-z 20922451 PMC3015030

[B98] Okumura H. (1970). Perilymph as a medium of oxygen-supply for the organ of corti. Archiv klin. Exp. Ohren Nasen Kehlkopfheilkunde 195, 257–265. 10.1007/BF00302953 5435960

[B99] Osako S. Hilding D. A. (1971). Electron microscopic studies of capillary permeability in normal and Ames waltzer deaf mice. Acta Otolaryngol. 71, 365–376. 10.3109/00016487109125376 5093631

[B100] Patterson C. E. Rhoades R. A. Garcia J. G. (1992). Evans blue dye as a marker of albumin clearance in cultured endothelial monolayer and isolated lung. J. Appl. Physiol. 72, 865–873. 10.1152/jappl.1992.72.3.865 1568982

[B101] Rabanel M. J. Aoun V. Elkin I. Mokhtar M. Hildgen P. (2012). Drug-loaded nanocarriers: passive targeting and crossing of biological barriers. Curr. Med. Chem. 19, 3070–3102. 10.2174/092986712800784702 22612696

[B102] Ruckenstein M. J. Hu L. (1999). Antibody deposition in the stria vascularis of the MRL-Faslpr mouse. Hear. Res. 127, 137–142. 10.1016/S0378-5955(98)00189-0 9925025

[B103] Ruckenstein M. J. Keithley E. M. Bennett T. Powell H. C. Baird S. Harris J. P. (1999a). Ultrastructural pathology in the stria vascularis of the MRL-Faslpr mouse. Hear. Res. 131, 22–28. 10.1016/S0378-5955(99)00018-0 10355601

[B104] Ruckenstein M. J. Milburn M. Hu L. (1999b). Strial dysfunction in the MRL-Faslpr mouse. Otolaryngol. Head Neck Surg. 121, 452–456. 10.1016/S0194-5998(99)70236-6 10504603

[B105] Sakagami M. Harada T. Sano M. Sakai S. Matsunaga T. (1987). Quantitative evaluation of pinocytosis of capillaries of the stria vascularis under normal and experimental conditions. Acta Otolaryngol. 103, 189–197. 10.3109/00016488709107272 3577750

[B106] Sakagami M. Matsunaga T. P. H. (1982b). Further fine structural observation on the permeability of capillaries in the stria vascularis and spiral ligament of the guinea pig. Ear Res. Jpn. 13, 39–42. 10.1007/BF00214780 7139688

[B107] Sakagami M. Matsunaga T. Hashimoto P. H. (1982a). Fine structure and permeability of capillaries in the stria vascularis and spiral ligament of the inner ear of the guinea pig. Cell Tissue Res. 226, 511–522. 10.1007/BF00214780 7139688

[B108] Salt A. N. Hirose K. (2018). Communication pathways to and from the inner ear and their contributions to drug delivery. Hear. Res. 362, 25–37. 10.1016/j.heares.2017.12.010 29277248 PMC5911243

[B109] Santi P. A. Lakhani B. N. Edwards L. B. Morizono T. (1985). Cell volume density alterations within the stria vascularis after administration of a hyperosmotic agent. Hear. Res. 18, 283–290. 10.1016/0378-5955(85)90045-0 3930457

[B110] Sato E. Shick H. E. Ransohoff R. M. Hirose K. (2010). Expression of fractalkine receptor CX3CR1 on cochlear macrophages influences survival of hair cells following ototoxic injury. J. Assoc. Res. Otolaryngol. 11, 223–234. 10.1007/s10162-009-0198-3 19936834 PMC2862920

[B111] Saunders N. R. Dziegielewska K. M. Møllgård K. Habgood M. D. (2015). Markers for blood-brain barrier integrity: how appropriate is Evans blue in the twenty-first century and what are the alternatives? Front. Neurosci. 9:385. 10.3389/fnins.2015.00385 26578854 PMC4624851

[B112] Schlageter K. E. Molnar P. Lapin G. D. Groothuis D. R. (1999). Microvessel organization and structure in experimental brain tumors: microvessel populations with distinctive structural and functional properties. Microvasc. Res. 58, 312–328. 10.1006/mvre.1999.2188 10527772

[B113] Schmiedt R. A. (1996). Effects of aging on potassium homeostasis and the endocochlear potential in the gerbil cochlea. Hear. Res. 102, 125–132. 10.1016/S0378-5955(96)00154-2 8951457

[B114] Schuknecht H. F. (1964). Further observations on the pathology of presbycusis. Arch. Otolaryngol. 80, 369–382. 10.1001/archotol.1964.00750040381003 14198699

[B115] Schuknecht H. F. Watanuki K. Takahashi T. Aziz Belal Jr A. Kimura R. S. Jones D. D.. (1974). Atrophy of the stria vascularis, a common cause for hearing loss. Laryngoscope 84, 1777–1821. 10.1288/00005537-197410000-00012 4138750

[B116] Schulte B. A. Schmiedt R. A. (1992). Lateral wall Na, K-ATPase and endocochlear potentials decline with age in quiet-reared gerbils. Hear. Res. 61, 35–46. 10.1016/0378-5955(92)90034-K 1326507

[B117] Schwartz I. McMenomey S. O. Russell N. J. Morton J. I. Trune D. R. (1992). Stria vascularis ultrastructural pathology in the C3H/lpr autoimmune strain mouse: a potential mechanism for immune-related hearing loss. Otolaryngol. Head Neck Surg. 106, 288–295. 10.1177/019459989210600317 1534162

[B118] Sekulic M. Puche R. Bodmer D. Petkovic V. (2023). Human blood-labyrinth barrier model to study the effects of cytokines and inflammation. Front. Mol. Neurosci. 16:1243370. 10.3389/fnmol.2023.1243370 37808472 PMC10551159

[B119] Sekulic-Jablanovic M. Paproth J. Sgambato C. Albano G. Fuster D. G. Bodmer D.. (2022). Lack of NHE6 and inhibition of NKCC1 associated with increased permeability in blood labyrinth barrier-derived endothelial cell layer. Front. Cell. Neurosci. 16:862119. 10.3389/fncel.2022.862119 35496913 PMC9039518

[B120] Shaddock L. C. Hamernik R. P. Wright C. G. (1985). A morphometric technique for analysis of cochlear vessels. Hear. Res. 20, 109–117. 10.1016/0378-5955(85)90162-5 4086378

[B121] Shi X. (2009a). Alteration of cochlear pericytes in response to noise trauma and the involvement of HIF-1α and VEGF. FASEB J. 23, 592–523. 10.1096/fasebj.23.1_supplement.592.23

[B122] Shi X. (2009b). Cochlear pericyte responses to acoustic trauma and the involvement of hypoxia-inducible factor-1α and vascular endothelial growth factor. Am. J. Pathol. 174, 1692–1704. 10.2353/ajpath.2009.080739 19349367 PMC2671258

[B123] Shi X. (2010). Resident macrophages in the cochlear blood-labyrinth barrier and their renewal via migration of bone-marrow-derived cells. Cell Tissue Res. 342, 21–30. 10.1007/s00441-010-1040-2 20838812

[B124] Shi X. (2023). Research advances in cochlear pericytes and hearing loss. Hear. Res. 438:108877. 10.1016/j.heares.2023.108877 37651921 PMC10538405

[B125] Shi X. Han W. Yamamoto H. Tang W. Lin X. Xiu R.. (2008). The cochlear pericytes. Microcirculation 15, 515–529. 10.1080/10739680802047445 19086261 PMC3647450

[B126] Spicer S. S. Schulte B. A. (2005a). Novel structures in marginal and intermediate cells presumably relate to functions of apical versus basal strial strata. Hear. Res. 200, 87–101. 10.1016/j.heares.2004.09.006 15668041

[B127] Spicer S. S. Schulte B. A. (2005b). Pathologic changes of presbycusis begin in secondary processes and spread to primary processes of strial marginal cells. Hear. Res. 205, 225–240. 10.1016/j.heares.2005.03.022 15953531

[B128] Stahle J. (1984). Medical treatment of fluctuant hearing loss in Meniere's disease. Am. J. Otol. 5, 529–533. 6393774

[B129] Steel K. P. Barkway C. (1989). Another role for melanocytes: their importance for normal stria vascularis development in the mammalian inner ear. Development 107, 453–463. 10.1242/dev.107.3.453 2612372

[B130] Strauss R. E. Gourdie R. G. (2020). Cx43 and the actin cytoskeleton: Novel roles and implications for cell-cell junction-based barrier function regulation. Biomolecules 10, 1656. 10.3390/biom10121656 33321985 PMC7764618

[B131] Sun W. Wang W. (2015). Advances in research on labyrinth membranous barriers. J. Otol. 10, 99–104. 10.1016/j.joto.2015.11.003 29937790 PMC6002577

[B132] Suzuki M. Sakamoto T. Kashio A. Yamasoba T. (2016). Age-related morphological changes in the basement membrane in the stria vascularis of C57BL/6 mice. Eur. Arch. Otorhinolaryngol. 273, 57–62. 10.1007/s00405-014-3478-4 25555607

[B133] Suzuki M. Yamasoba T. Ishibashi T. Miller J. M. Kaga K. (2002). Effect of noise exposure on blood–labyrinth barrier in guinea pigs. Hear. Res. 164, 12–18. 10.1016/S0378-5955(01)00397-5 11950520

[B134] Suzuki T. Takamatsu T. Oyamada M. (2003). Expression of gap junction protein connexin43 in the adult rat cochlea: comparison with connexin26. J. Histochem. Cytochem. 51, 903–912. 10.1177/002215540305100705 12810840

[B135] Takeuchi S. Ando M. (1998). Dye-coupling of melanocytes with endothelial cells and pericytes in the cochlea of gerbils. Cell Tissue Res. 293, 271–275. 10.1007/s004410051118 9662649

[B136] Takeuchi S. Ando M. Sato T. Kakigi A. (2001). Three-dimensional and ultrastructural relationships between intermediate cells and capillaries in the gerbil stria vascularis. Hear. Res. 155, 103–112. 10.1016/S0378-5955(01)00252-0 11335080

[B137] Taukulis I. A. Olszewski R. T. Korrapati S. Fernandez K. A. Boger E. T. Fitzgerald T. S.. (2021). Single-cell RNA-seq of cisplatin-treated adult stria vascularis identifies cell type-specific regulatory networks and novel therapeutic gene targets. Front. Mol. Neurosci. 14:718241. 10.3389/fnmol.2021.718241 34566577 PMC8458580

[B138] Thomopoulos G. N. Spicer S. S. Gratton M. A. Schulte B. A. (1997). Age-related thickening of basement membrane in stria vascularis capillaries. Hear. Res. 111, 31–41. 10.1016/S0378-5955(97)00080-4 9307309

[B139] Thulasiram M. R. Ogier J. M. Dabdoub A. (2022). Hearing function, degeneration, and disease: Spotlight on the stria vascularis. Front. Cell Dev. Biol. 10:841708. 10.3389/fcell.2022.841708 35309932 PMC8931286

[B140] Tornabene S. V. Sato K. Pham L. Billings P. Keithley E. M. (2006). Immune cell recruitment following acoustic trauma. Hear. Res. 222, 115–124. 10.1016/j.heares.2006.09.004 17081714

[B141] Trune D. R. (1997). Cochlear immunoglobulin in the C3H/lpr mouse model for autoimmune hearing loss. Otolaryngol. Head Neck Surg. 117, 504–508. 10.1016/S0194-59989770022-6 9374175

[B142] Trune D. R. (2002). “Mouse models for immunologic diseases of the auditory system,” in Handbook of Mouse Auditory Research: From Behavior to Molecular Biology, ed Willott J. F. (New York, NY: CRC Press), 505–531.

[B143] Trune D. R. Craven J. P. Morton J. I. Mitchell C. (1989). Autoimmune disease and cochlear pathology in the C3H/lpr strain mouse. Hear. Res. 38, 57–66. 10.1016/0378-5955(89)90128-7 2708160

[B144] Trune D. R. Kempton J. B. (2001). Aldosterone and prednisolone control of cochlear function in MRL/MPJ-Faslpr autoimmune mice. Hear. Res. 155, 9–20. 10.1016/S0378-5955(01)00240-4 11335072

[B145] Úlehlová L. (1983). Stria vascularis in acoustic trauma. Arch. Otorhinolaryngol. 237, 133–138. 10.1007/BF00463612 6847511

[B146] Veiga M. Kuhweide R. Demaerel V. De Pauw R. De Foer B. Casselman J. W. (2021). Labyrinthine enhancement on 3D black blood MR images of the brain as an imaging biomarker for cisplatin ototoxicity in (lung) cancer patients. Neuroradiology 63, 81–90. 10.1007/s00234-020-02504-x 32761280

[B147] Wajant H. (2002). The Fas signaling pathway: more than a paradigm. Science 296, 1635–1636. 10.1126/science.1071553 12040174

[B148] Wangemann P. Marcus D.C. (2017). “Ion and fluid homeostasis in the Cochlea,” in Understanding the Cochlea. Springer Handbook of Auditory Research, Vol. 62, eds Manley G. Gummer A. Popper A. Fay R. (Cham: Springer).

[B149] Watanabe K. Tanaka Y. (1997). Horseradish peroxidase permeation from the capillaries of the stria vascularis after inoculation of endotoxin into the middle ear. Ann. Otol. Rhinol. Laryngol. 106, 394–398. 10.1177/000348949710600507 9153104

[B150] Watanabe K. I. Jinnouchi K. Hess A. Michel O. Yagi T. (2001). Detection of apoptotic change in the lipopolysaccharide (LPS)-treated cochlea of guinea pigs. Hear. Res. 158, 116–122. 10.1016/S0378-5955(01)00291-X 11506943

[B151] Williamson J. R. Vogler N. J. Kilo C. (1973). “Early capillary basement membrane changes in subjects with diabetes mellitus,” in Vascular and Neurological Changes in Early Diabetes, eds Camerini-Dávalos R. A. Cole H. S. (Academic Press), 363–371. 10.1016/b978-0-12-027362-1.50044-3 4720373

[B152] Wood J. W. Bas E. Gupta C. Selman Y. Eshraghi A. Telischi F. F.. (2014). Otoprotective properties of mannitol against gentamicin induced hair cell loss. Otol. Neurotol. 35, e187–e19 10.1097/MAO.0000000000000342 24662629

[B153] Wright C. G. Lee D. H. (1989). Pigmented cells of the stria vascularis and spiral ligament of the chinchilla. Acta Otolaryngol. 108, 190–200. 10.3109/00016488909125518 2479217

[B154] Wu Y. X. Zhu G. X. Liu X. Q. Sun F. Zhou K. Wang S.. (2014). Noise alters guinea pig's blood-labyrinth barrier ultrastructure and permeability along with a decrease of cochlear Claudin-5 and Occludin. BMC Neurosci. 15, 1–10. 10.1186/s12868-014-0136-0 25539640 PMC4299297

[B155] Xu R. D. Watanabe K. Komatsuzaki A. (1994). Permeability for horseradish peroxidase in strial capillaries in each turn of cochlea. ORL 56, 183–189. 10.1159/000276653 8078670

[B156] Yoshida M. Uemura T. (1991). Effect of glycerol and mannitol on perilymphatic PO2 in guinea pig cochlea. Otolaryngol. Head Neck Surg. 104, 495–498. 10.1177/019459989110400412 1903862

[B157] Yu W. Zong S. Du P. Zhou P. Li H. Wang E.. (2021). Role of the stria vascularis in the pathogenesis of sensorineural hearing loss: a narrative review. Front. Neurosci. 15:774585. 10.3389/fnins.2021.774585 34867173 PMC8640081

[B158] Yu W. Zong S. Zhou P. Wei J. Wang E. Ming R.. (2022). Cochlear marginal cell pyroptosis is induced by cisplatin via NLRP3 inflammasome activation. Front. Immunol. 13:823439. 10.3389/fimmu.2022.823439 35529876 PMC9067579

[B159] Zhang F. Dai M. Neng L. Zhang J. H. Zhi Z. Fridberger A.. (2013). Perivascular macrophage-like melanocyte responsiveness to acoustic trauma—a salient feature of strial barrier associated hearing loss. FASEB J. 27:3730. 10.1096/fj.13-232892 23729595 PMC3752533

[B160] Zhang J. Chen S. Hou Z. Cai J. Dong M. Shi X. (2015). Lipopolysaccharide-induced middle ear inflammation disrupts the cochlear intra-strial fluid–blood barrier through down-regulation of tight junction proteins. PLoS ONE 10:e0122572. 10.1371/journal.pone.0122572 25815897 PMC4376743

[B161] Zhang J. Hou Z. Wang X. Jiang H. Neng L. Zhang Y.. (2021). VEGFA165 gene therapy ameliorates blood-labyrinth barrier breakdown and hearing loss. JCI Insight 6:e143285. 10.1172/jci.insight.143285 33690221 PMC8119217

[B162] Zhang J. Wang X. Hou Z. Neng L. Cai J. Zhang Y.. (2020a). Suppression of connexin 43 leads to strial vascular hyper-permeability, decrease in endocochlear potential, and mild hearing loss. Front. Physiol. 11:974. 10.3389/fphys.2020.00974 32922309 PMC7457066

[B163] Zhang N. Cai J. Xu L. Wang H. Liu W. (2020b). Cisplatin-induced stria vascularis damage is associated with inflammation and fibrosis. Neural Plast. 2020:8851525. 10.1155/2020/8851525 33029120 PMC7527906

[B164] Zhang W. Dai M. Fridberger A. Hassan A. DeGagne J. Neng L.. (2012). Perivascular-resident macrophage-like melanocytes in the inner ear are essential for the integrity of the intrastrial fluid–blood barrier. Proc. Nat. Acad. Sci. U. S. A. 109, 10388–10393. 10.1073/pnas.1205210109 22689949 PMC3387119

[B165] Zhang W. Xie J. Liu H. Wang M. (2023). Blood–labyrinth barrier breakdown in Meniere's disease. Eur. Arch. Otorhinolaryngol. 1–6. 10.1007/s00405-023-08353-7 38057488

[B166] Zidanic M. Brownell W. E. (1990). Fine structure of the intracochlear potential field. I. The silent current. Biophys. J. 57, 1253–1268. 10.1016/S0006-3495(90)82644-8 2393707 PMC1280835

[B167] Zlokovic B. V. Skundric D. S. Segal M. B. Lipovac M. N. Mackic J. B. Davson H. (1990). A saturable mechanism for transport of immunoglobulin G across the blood-brain barrier of the guinea pig. Exp. Neurol. 107, 263–270. 10.1016/0014-4886(90)90144-H 1689666

